# Synthesis and Characterization of Solvated Lanthanide(II) Bis(triisopropylsilyl)phosphide Complexes

**DOI:** 10.1021/acs.inorgchem.4c03135

**Published:** 2024-10-18

**Authors:** Jack Baldwin, Adam Brookfield, George F. S. Whitehead, Louise S. Natrajan, Eric J. L. McInnes, Meagan S. Oakley, David P. Mills

**Affiliations:** Department of Chemistry, The University of Manchester, Oxford Road, Manchester M13 9PL, U.K.

## Abstract

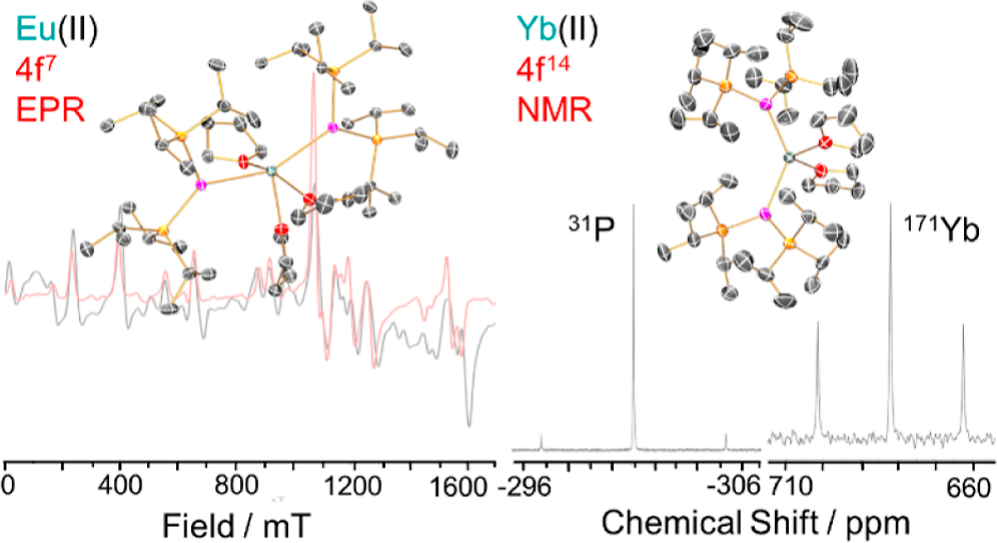

Lanthanide (Ln) silylamide chemistry is well-developed, but the corresponding silylphosphide chemistry is immature; there are only ten structurally characterized examples of Ln(II) bis(trimethylsilyl)phosphide complexes to date and no reported derivatives with bulkier R-groups. Here, we report the synthesis of the first f-block bis(triisopropylsilyl)phosphide complexes, [Ln{P(Si^*i*^Pr_3_)_2_}_2_(THF)_*x*_] (**1-Ln**; Ln = Sm, Eu, *x* = 3; Ln = Yb, *x* = 2), by the respective salt metathesis reactions of parent [LnI_2_(THF)_2_] with 2 equiv of [Na{P(Si^*i*^Pr_3_)_2_}]_*n*_ in toluene. Complexes **1-Ln** were characterized by a combination of NMR, EPR, ATR–IR, electronic absorption and emission spectroscopies, elemental analysis, SQUID magnetometry, and single crystal X-ray diffraction. These data contrast with those obtained for related Ln(II) bis(trimethylsilyl)phosphide complexes due to the bulkier ligands in **1-Ln** and also with Ln(II) bis(triisopropylsilyl)amide complexes due to a combination of longer Ln–P vs. Ln–N bonds and the softer nature of P- vs. N-donor ligands.

## Abstract 2

We report the synthesis of the first f-block bis(triisopropylsilyl)phosphide complexes, [Ln{P(Si^*i*^Pr_3_)_2_}_2_(THF)_*x*_] (Ln = Sm, Eu, *x* = 3; Ln = Yb, *x* = 2) and their characterization by a combination of experimental and computational studies.

## Introduction

Lanthanide (Ln) compounds have unique magnetic, optical, and catalytic properties, which have been exploited in a wide range of technological applications.^1^ Ln bonding regimes are predominantly ionic and therefore lack directionality, but Ln coordination spheres can be controlled by using appropriate ligands and anaerobic environments.^2,3^ Design criteria for Ln complexes with ideal structural features for enhanced physiochemical properties have been developed.^4−9^ However, due to Ln ions being hard Lewis acids and their preferential binding to hard Lewis bases such as nitrogen, oxygen, and halides, there is a paucity of Ln complexes containing softer p-block donor atom ligands.^1^ This is exemplified by the pnictogen series, where there are a large number of structurally authenticated examples of Ln amide complexes,^10−13^ but there are few heavy group 15 analogues in comparison.^14−16^

Over the past half a century, bis(trimethylsilyl)amide, {N(SiMe_3_)_2_} (N″), has provided some of the most fascinating advances in Ln chemistry since the first reports of the landmark trigonal pyramidal Ln(III) complexes [Ln{N(SiMe_3_)_2_}_3_].^10,17,18^ Seminal examples of Ln(II) N″ complexes include the first structurally characterized Eu(II) amide complexes [Eu{N(SiMe_3_)_2_}_2_(sol)_2_] (sol = DME or THF)^19^ and a rare Sc(II) complex [K(2.2.2-cryptand)][Sc{N(SiMe_3_)_2_}_3_].^20^ Although not as well-established, the larger bis(triisopropylsilyl)amide ligand, {N(Si^*i*^Pr_3_)_2_}, has also provided notable Ln(II) complexes, such as the near-linear examples [Ln{N(Si^*i*^Pr_3_)_2_}_2_] (Ln = Sm, Eu, Tm, Yb).^21,22^ In contrast, only a handful of f-element bis(trimethylsilyl)phosphide ({P(SiMe_3_)_2_}, P″) complexes have been structurally authenticated to date,^14,23,24^ and the only Ln(II) examples are [Sm{P(SiMe_3_)_2_}{μ-P(SiMe_3_)_2_}_3_Sm(THF)_3_],^25^ [{Ln[P(SiMe_3_)_2_]_3_(THF)}_2_(μ-I)K_3_(THF)] (Ln = Sm, Eu), [KYb{P(SiMe_3_)_2_}_3_{μ-K[P(SiMe_3_)_2_]}_2_]_∞_, *trans*-[Ln{P(SiMe_3_)_2_}_2_(py)_4_] (Ln = Sm, Eu, Yb; py = pyridine) and [Ln{P(SiMe_3_)_2_}_2_(18-crown-6)] (Ln = Sm, Eu, Yb).^24^ Conversely, Ln(II) phosphides with other substituents are well-known,^14^ e.g. [Yb(PPh_2_)_2_(THF)_4_],^26^ [Ln{P(Mes)_2_}_2_(THF)_4_] (Ln = Sm, Yb; Mes = C_6_H_2_Me_3_-2,4,6),^27,28^ [Ln(PPh_2_)_2_(*N*-methylimidazole)_4_] (Ln = Eu, Yb),^29,30^ [Ln{(μ-P^*t*^Bu_2_)_2_Li(THF)}_2_] (Ln = Sm, Eu, Yb),^31−33^ [Ln{HP(Mes*)}_2_(THF)_4_] (Ln = Eu, Yb; Mes* = C_6_H_2_^*t*^Bu_3_-2,4,6),^34^ [Ln{P[CH(SiMe_3_)_2_](C_6_H_4_-2-CH_2_NMe_2_)}_2_(THF)_*n*_] (Ln = Sm, *n* = 1; Ln = Yb, *n* = 0),^35^ [Sm{P[CH(SiMe_3_)_2_](Ar)}_2_(DME)(THF)] (Ar = Ar′ = C_6_H_3_-2-OMe-3-Me, *n* = 0; Ar = Ar” C_6_H_4_-2-OMe, *n* = 1),^35^ [Yb{P[CH(SiMe_3_)_2_](Ar′)}_2_(THF)_2_],^36^ and [Yb{P[CH(SiMe_3_)_2_](C_6_H_4_-2-O)}(THF)]_4_.^36^

Conversely, to date, there are no reported examples of f-element complexes containing bis(triisopropylsilyl)phosphide, {P(Si^*i*^Pr_3_)_2_}. While H{N(Si^*i*^Pr_3_)_2_} and its alkali metal salts can be easily synthesized from commercially available reagents starting from NH_3_^21^ or NaNH_2_,^37^ HP(Si^*i*^Pr_3_)_2_ was previously only isolated as a minor byproduct in 13% yield from the salt metathesis reaction of [Li(PH_2_)(DME)] with ClSi^*i*^Pr_3_;^38^ the THF-solvated lithium salt, [Li{μ-P(Si^*i*^Pr_3_)_2_}(THF)]_2_, was prepared by the deprotonation of HP(Si^*i*^Pr_3_)_2_ with ^*n*^BuLi in THF.^39^ Further to this, [Li(PH_2_)(DME)] was synthesized from highly toxic gaseous PH_3_,^40^ and a synthetic route from more readily available red phosphorus has not yet been disclosed; this advance has allowed the chemistry of the smaller phosphide P″ to be developed more rapidly (see above), though this still typically involves the large-scale synthesis of P(SiMe_3_)_3_ via PNa_3_, which requires specialist glassware and protocols.^41^

Here, we report the synthesis of the first structurally authenticated Ln(II) {P(Si^*i*^Pr_3_)_2_} complexes, [Ln{P(Si^*i*^Pr_3_)_2_}_2_(THF)_*x*_] (**1-Ln**; Ln = Sm, Eu, *x* = 3; Ln = Yb, *x* = 2). These complexes are characterized by a combination of NMR, EPR, ATR–IR, electronic absorption and emission spectroscopies, elemental analysis, SQUID magnetometry, and single crystal X-ray diffraction. We find that the data obtained for **1-Ln** complement and contrast with those previously reported for related Ln(II) P″ and {N(Si^*i*^Pr_3_)_2_} complexes due to differences in ligand steric requirements and hard and soft acid and base (HSAB) considerations.

## Results and Discussion

### Synthesis

**Scheme 1:**
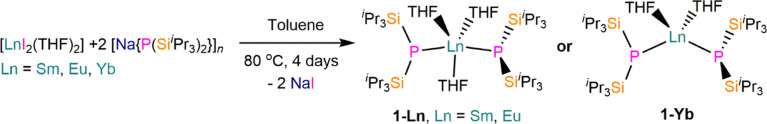
Synthesis of **1-Ln**

By adapting a combination of literature procedures for the synthesis of P(SiMe_3_)_3_ from PNa_3_ with 3 equiv of ClSiMe_3_,^41^ the salt metathesis reaction of PNa_3_ (prepared in situ from red phosphorus, sodium metal and naphthalene) with 3 equiv of ClSi^*i*^Pr_3_ in DME at reflux for 24 h gave [Na{P(Si^*i*^Pr_3_)_2_}]_*n*_ as a white powder in 45% crude yield following workup; these improved yields were obtained by following the procedures of Kays and co-workers, as our original preparation only gave yields of ca. 10%.^42^ A small portion of the solid was recrystallized from a saturated DME solution to give crystals of the solvated adduct, [Na{P(Si^*i*^Pr_3_)_2_}(DME)_2_]. This method represents a convenient entry point to {P(Si^*i*^Pr_3_)_2_} coordination chemistry starting from red phosphorus, where previously PH_3_ was used as a starting material (see above).^40^ The separate salt metathesis reactions of [LnI_2_(THF)_2_] (Ln = Sm, Eu, Yb) with 2 equiv of [Na{P(Si^*i*^Pr_3_)_2_}]_*n*_ in toluene heated at 80 °C for 4 days gave complexes **1-Ln** (Ln = Sm, Eu, Yb) in 43–64% yields following recrystallization from pentane solutions containing several drops of THF (Scheme 1). We attempted to synthesize an analogous Tm(II) complex **1 Tm** using similar methods, whereby several drops of THF were added to a mixture of TmI_2_ and 2 equiv of [Na{P(Si^*i*^Pr_3_)_2_}]_*n*_ in toluene at −30 °C; this temperature was maintained for 3 h before warming to room temperature and then following the protocols above. We were unable to isolate a Tm-containing product by this approach, which we attribute to the tendency of more reducing Tm(II) centers to react with ethereal solvents such as THF to give Tm(III) products.^1^ A Tm(II) {P(Si^*i*^Pr_3_)_2_} complex should be isolable, but a different synthetic approach is needed, which would require a separate dedicated study.

The bulk compositions of microcrystalline [Na{P(Si^*i*^Pr_3_)_2_}]_*n*_ and **1-Ln** were assessed by a combination of elemental analysis and ATR–IR spectroscopy (ATR–IR spectra are compiled in Supporting Information Figures S1–S5). We find that for **1-Ln** elemental analysis, results are generally in good agreement with predicted values, but the carbon values obtained were reproducibly lower than expected; we attribute this observation to incomplete combustion arising from carbide formation,^43,44^ which is a common feature for analogous Ln {N(Si^*i*^Pr_3_)_2_} and Ln {P(SiMe_3_)_2_} complexes.^21,22,45,46^

### X-ray Crystallography

**Figure 1:**
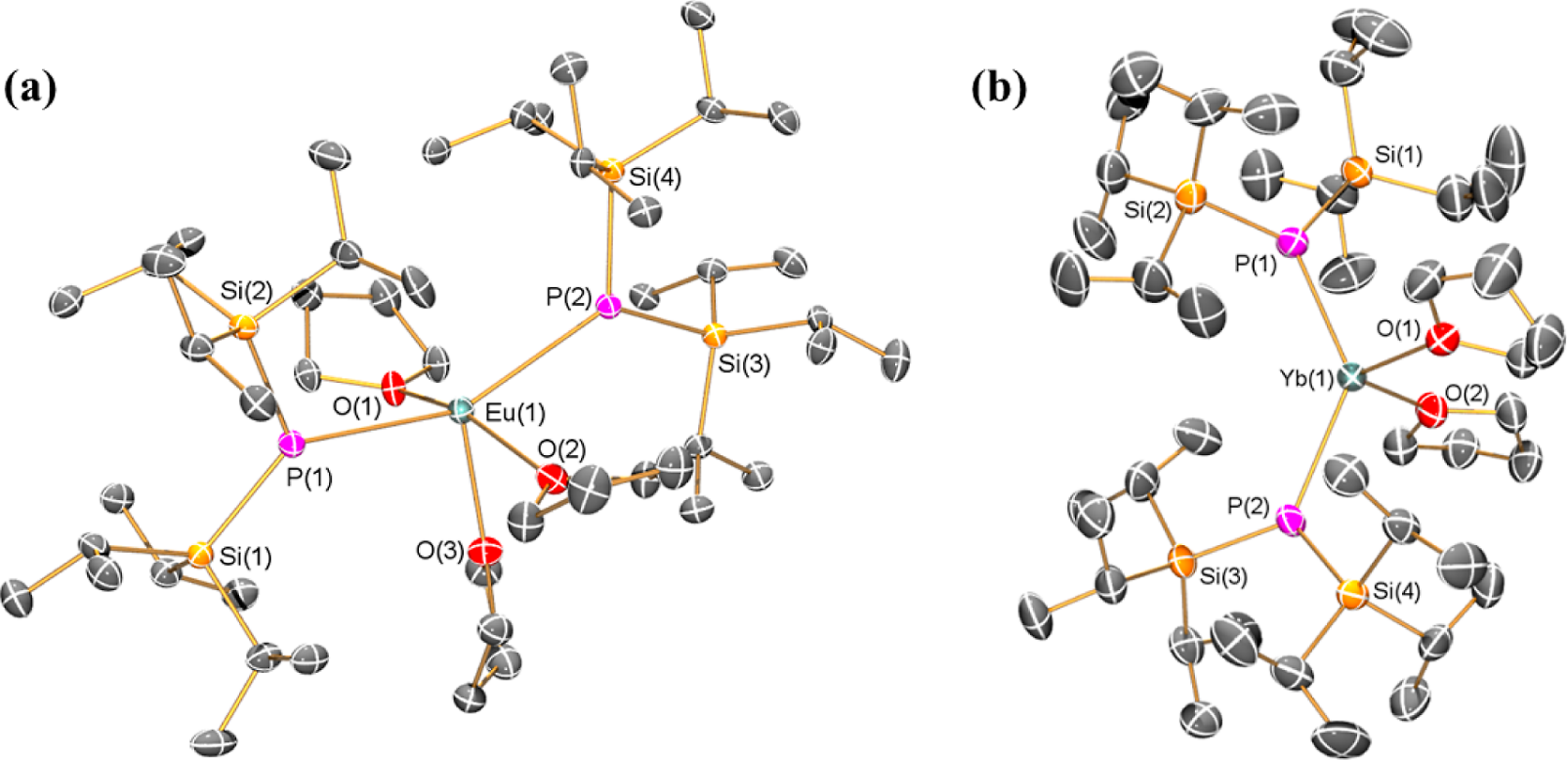
Solid-state structures of (a) **1-Eu** and (b) **1-Yb**, with selected atomic labeling; Ln = cyan, P = magenta, Si = yellow, O = red, C = gray. Displacement ellipsoids set at 50% probability level, hydrogen atoms omitted for clarity.

The solid state structures of **1-Ln** were determined by single crystal XRD (see Figure 1 for depictions of **1-Eu** and **1-Yb**; [Na{P(Si^*i*^Pr_3_)_2_}(DME)_2_] is shown in the Supporting Information Figure S6, and as **1-Sm** is isostructural to **1-Eu**, it is depicted in Figure S7. Selected bond distances and angles for all complexes are presented in Table 1, and crystallographic parameters are compiled in Table S1). The Ln(II) ions in the five-coordinate complexes **1-Sm** and **1-Eu** are bound by two {P(Si^*i*^Pr_3_)_2_} and three THF ligands in a geometry that is intermediate between trigonal bipyramidal and square-based pyramidal, while **1-Yb** has one less THF bound and is distorted tetrahedral (P–Ln–P angles: **1-Sm**: 156.11(3)°, **1-Eu**: 156.10(3)°, **1-Yb**: 133.48(3)°); the lower coordination number in **1-Yb** is due to its smaller size (7-coordinate Ln(II) ionic radii: Sm(II), 1.22 Å; Eu(II), 1.20 Å; Yb(II), 1.08 Å).^47^ These geometries were quantified by Shape2.0 (see Supporting Information Tables S3 and S4), which generates numerical values for the fits of atomic coordinates of the ligand donor atoms to all possible ideal three-dimensional shapes; the lower the value of these shape indices, the closer the fit to a particular shape.^48^ For **1-Sm** (3.728) and **1-Eu** (3.718), the lowest value shape indices correspond to square-based pyramidal geometries and **1-Yb** (3.311) is best-described as tetrahedral. The distortion of the coordination spheres in five-coordinate complexes can be gauged by the geometric parameter τ_5_ = (β – α)/60, where β and α are the largest and second-largest angles in the coordination sphere, respectively.^49^ The τ_5_ parameter quantifies the degree of trigonality within the structural continuum between square-based pyramidal (τ_5_ = 0) and trigonal bipyramidal (τ_5_ = 1), and the τ_5_ values of **1-Sm** (0.8535) and **1-Eu** (0.8560) are closer to the latter (see Supporting Information Table S4). Similarly, four-coordinate systems can be defined by the geometric parameter τ_4_ = [360 – (α + β)]/141, which quantifies the degree of tetrahedrality between square planar (τ_4_ = 0) and tetrahedral (τ_4_ = 1).^50^ For **1-Yb**, the complex is distorted from an ideal tetrahedral geometry with a τ_4_ value of 0.7976 (see Supporting Information Table S4).

**Table 1:** Selected Bond Lengths and Angles for **1-Ln**

parameter	**1-Sm**	**1-Eu**	**1-Yb**
Ln(1)–P(1)/Å	3.0216(10)	3.0107(13)	2.8001(10)
Ln(1)–P(2)/Å	3.0455(8)	3.0366(12)	2.8128(9)
Ln(1)–O(1)/Å	2.553(2)	2.542(4)	2.356(3)
Ln(1)–O(2)/Å	2.556(2)	2.545(4)	2.400(2)
Ln(1)–O(3)/Å	2.516(2)	2.495(4)	
P(1)–Ln(1)–P(2)/°	156.11(3)	156.10(3)	133.48(3)
O(1)–Ln(1)–O(2)/°	160.08(7)	160.22(12)	99.91(9)
O(1)–Ln(1)–O(3)/°	80.12(7)	80.74(12)	
O(2)–Ln(1)–O(3)/°	80.56(7)	80.05(12)	
P(1)–Ln(1)–O(1)/°	93.95(5)	93.66(8)	103.13(8)
P(1)–Ln(1)–O(2)/°	93.81(5)	94.05(9)	103.93(7)
P(1)–Ln(1)–O(3)/°	98.94(6)	99.32(15)	
P(2)–Ln(1)–O(1)/°	91.73(5)	88.58(8)	96.54(7)
P(2)–Ln(1)–O(2)/°	88.55(5)	91.69(8)	113.68(7)
P(2)–Ln(1)–O(3)/°	104.90(6)	104.52(9)	
Σ angles about P/°	359.71(8)	359.72(11)	358.28(9)
	359.99(8)	359.99(11)	359.16(8)

The mean Ln–P distances in **1-Ln** (Ln = Sm, 3.0336(13) Å; Eu, 3.024(2) Å; Yb, 2.8065(13) Å) have the expected differences from ionic radii considerations (see above). The Ln–P bond lengths in **1-Ln** are comparable with the respective Ln–P distances seen in [Sm{P(SiMe_3_)_2_}{μ-P(SiMe_3_)_2_}_3_Sm(THF)_3_] (Sm–P_terminal_ = 3.027(3) Å; Sm–P_bridging_ range = 3.100(3)–3.178(3) Å),^23^ [{Ln[P(SiMe_3_)_2_]_3_(THF)}_2_(μ-I)K_3_(THF)] (Ln = Sm, 2.993(2)–3.068(2) Å; Eu, 2.988(2)–3.060(2) Å),^24^ [Ln{P(SiMe_3_)_2_}_2_(18-crown-6)] (Ln = Sm, 3.047(6)–3.126(7) Å; Eu, 3.075(3)–3.099(3) Å),^24^ and [Ln{P(SiMe_3_)_2_}_2_(py)_4_] (Ln = Sm, 3.0342(9) Å; Eu, 3.0364(7) Å).^24^ However, in **1-Yb**, the Yb–P distances are shorter than the Yb–P bonds found in the related Yb(II) phosphide complexes [Yb(PPh_2_)_2_(THF)_4_] (2.991(2) Å),^26^ [Yb{P[CH(SiMe_3_)_2_](Ar′)}_2_(THF)_2_] (2.969(3) Å),^36^ [Yb{P(SiMe_3_)_2_}_2_(py)_4_] (2.9109(10)–2.9358(9) Å), and [Yb{P(SiMe_3_)_2_}_2_(18-crown-6)] (2.9662(10) Å).^24^ The mean Ln–O distances in **1-Ln** (Ln = Sm, 2.542(3) Å; Eu, 2.527(8) Å; Yb, 2.378(4) Å) all fall within the range of known Ln–O_THF_ bond lengths (2.284(13)–2.951(3) Å for Sm, 2.364(4)–2.811(4) Å for Eu, and 2.167(4)–2.610(3) Å for Yb);^51^ those of **1-Sm** are shorter than seen for [Sm{P(SiMe_3_)_2_}{μ-P(SiMe_3_)_2_}_3_Sm(THF)_3_] (2.593(13) Å mean),^25^ and those of **1-Yb** are shorter than observed in both [Yb(PPh_2_)_2_(THF)_4_] (2.436(5) Å mean)^26^ and [Yb{P[CH(SiMe_3_)_2_](Ar′)}_2_(THF)_2_] (2.534(6) Å mean).^36^ We attribute the relatively short Yb–P and–O bond distances in **1-Yb** to the electronically and sterically unsaturated Yb(II) ions, arising from a low metal coordination number.

The geometry of the phosphorus atoms in **1-Ln** are close to ideal trigonal planar, as defined by the sum of the angles about the central phosphorus atom (**1-Sm**, 359.71(8) and 359.99(8)°; **1-Eu**, 359.72(11) and 359.99(11)°; **1-Yb**, 358.28(9) and 359.16(8)°). This differs from the trigonal pyramidal phosphorus geometries in many other Ln(II) bis-phosphide complexes, such as [Sm{P(SiMe_3_)_2_}{μ-P(SiMe_3_)_2_}_3_Sm(THF)_3_] (326.6(2)°),^25^ [Ln{P(SiMe_3_)_2_}_2_(py)_4_] (Ln = Sm, 344.66(9)°; Eu, 344.52(7)°),^24^ [Yb(PPh_2_)_2_(THF)_4_] (332.65(10)°),^26^ and [Yb{P[CH(SiMe_3_)_2_](Ar′)}_2_(THF)_2_] (341.7(7)°).^36^ However, some Ln(II) silylphosphide complexes have exhibited nearly planar phosphorus geometries, such as [Ln{P(SiMe_3_)_2_}_2_(18-crown-6)] (Ln = Sm, 352.6(5) and 354.0(7)°; Eu, 352.1(3) and 353.9(3)°),^24^ [Yb{P(SiMe_3_)_2_}_2_(py)_4_] (359.93(7)°),^24^ and [KYb{P(SiMe_3_)_2_}_3_{μ-K[P(SiMe_3_)_2_]}_2_]_∞_ (357.5(2) and 358.3(6)°).^24^ Although the trigonal planar phosphorus geometries in **1-Ln** could be interpreted as the filled P 3p orbital interacting with the Ln(II) center, this is solely attributed to steric effects due to the electrostatics-dominated bonding regimes in Ln complexes.^1^

### Solution NMR Spectroscopy

^1^H, ^13^C{^1^H}, and ^29^Si DEPT90 and ^31^P{^1^H} NMR spectra were collected for **1-Ln**, and ^171^Yb{^1^H} NMR spectra were also obtained for diamagnetic **1-Yb** (see Supporting Information Figures S7–S21 for NMR spectra of all complexes; selected NMR data are compiled in Table 2). Due to the paramagnetism of Sm(II) and Eu(II) ions, significant paramagnetic broadening and shifting of the resonances in the NMR spectra of **1-Sm** and **1-Eu** were evident, and we were not able to fully interpret these data. In the ^1^H NMR spectrum of **1-Sm**, the methine (δ_H_: 1.15 ppm) and methyl (δ_H_: 1.29 ppm) resonances of the isopropyl groups were assigned by their relative integrations, but we were not able to extract coupling constants due to low resolution; two broad resonances at −0.90 and 5.68 ppm were similarly assigned to the α- and β-methylene protons of the three bound THF molecules. In the ^13^C{^1^H} NMR spectrum of **1-Sm**, two resonances were also observed for the methine (δ_C_: 24.98 ppm) and methyl (δ_C_: 28.78 ppm) groups, though we did not observe the α- and β-positions of THF due to paramagnetic broadening. We were not able to confidently assign any resonance to **1-Sm** in the ^29^Si DEPT90 NMR spectrum, with only a diamagnetic impurity being observed at δ_Si_ = −20.49 ppm; similarly, we were not able to observe resonances corresponding to **1-Sm** in ^31^P{^1^H} NMR spectra collected between +1000 and −1000 ppm, with a singlet at δ_P_ = −286.50 ppm assigned to a minor impurity of [Na{P(Si^*i*^Pr_3_)_2_}]_*n*_. The NMR spectra of **1-Eu** exhibit even more pronounced paramagnetic broadening, such that we were even not able to interpret the ^1^H NMR spectrum, though a distinctive resonance was seen at δ_H_ = 1.26 ppm.

**Table 2:** ^1^H, ^13^C{^1^H}, ^29^Si DEPT90, ^31^P{^1^H}, and ^171^Yb{^1^H} NMR Data for **1-Sm** and **1-Yb** in C_6_D_6_

complex	^1^H (δ)	^13^C{^1^H} (δ)	^29^Si DEPT90 (δ)	^31^P{^1^H} (δ)	^171^Yb{^1^H} (δ)
**1-Sm**	–0.90 (br, 12H, THF)1.15 (br, 12H, ^*i*^Pr C*H*)1.29 (br, 72H, ^*i*^Pr C*H*_3_)5.68 (br, 12H, THF)	24.98 (s, ^*i*^Pr *C*H)28.78 (s, ^*i*^Pr *C*H_3_)THF–C*H*_2_ not observed			
**1-Yb**	1.41 (br, 92H, ^*i*^Pr C*H*_3_, ^*i*^Pr C*H* and THF–C*H*_2_) 3.85 (br, 8H, THF–C*H*_2_)	16.90 (Vir. t, ^*i*^Pr *C*H, ^2^*J*_PC_ = 10.3 Hz) 20.57 (Vir. t, ^*i*^Pr *C*H_3_, ^3^*J*_PC_ = 3.6 Hz)	24.30 (Vir. t, P–*Si*, ^1^*J*_PSi_ = 15.8 Hz)	–301.10 (s, *P*–Yb, ^1^*J*_YbP_ = 1382.1 Hz)	682 (t, *Yb*–P, ^1^*J*_YbP_ = 1382.9 Hz)

**Figure 2:**
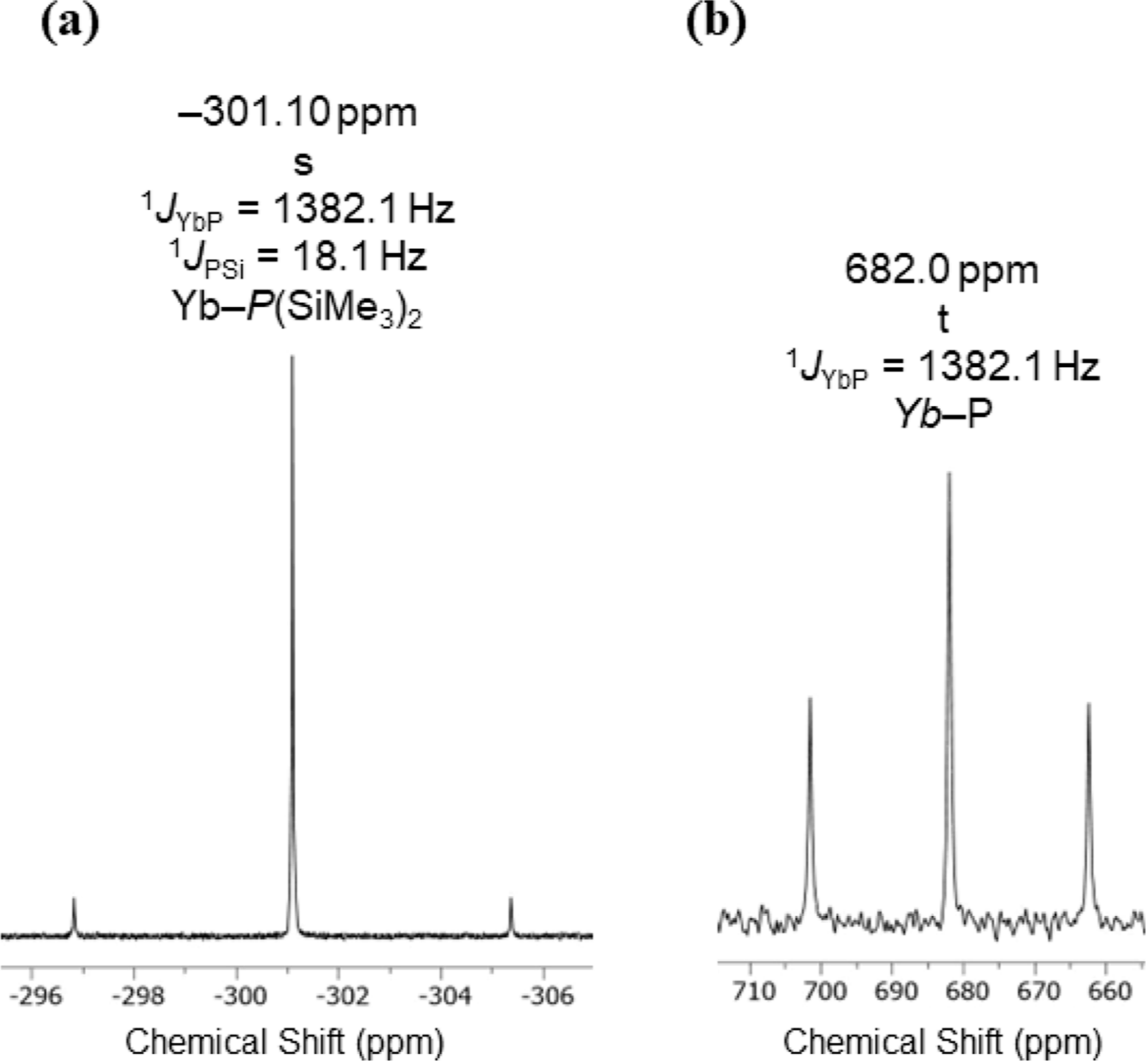
(a) ^31^P{^1^H} and (b) ^171^Yb{^1^H} NMR spectra of **1-Yb** in C_6_D_6_ at 162 and 71 MHz, respectively.

By contrast, all NMR spectra of diamagnetic **1-Yb** could be confidently assigned, though the resonances in the ^1^H NMR spectra are relatively broad, precluding the extraction of coupling constants. The methyl and methine carbon atoms of the ^*i*^Pr groups present as virtual triplets in the ^13^C{^1^H} NMR spectrum of **1-Yb** due to the strongly coupled P atoms, (δ_Si_ = 16.90 ppm, ^2^*J*_PC_ = 10.3 Hz, ^*i*^Pr-*C*H; δ_Si_ = 20.57 ppm, ^3^*J*_PC_ = 3.6 Hz, ^*i*^Pr-*C*H_3_), while a virtual triplet is seen in the ^29^Si DEPT90 NMR at δ_Si_ = 24.30 ppm (^1^*J*_PSi_ = 15.8 Hz) from the same second-order effects. The ^31^P{^1^H} NMR spectrum of **1-Yb** exhibits a signal at −301.10 ppm, with satellites to 14.3% abundant *I* = 1/2 ^171^Yb nuclei giving ^1^*J*_YbP_ = 1382 Hz; coupling between the ^31^P and 4.67% abundant *I* = 1/2 ^29^Si nuclei is also observed with ^1^*J*_PSi_ ≈ 18 Hz (Figure 2a). This ^31^P chemical shift value lies downfield to that reported previously for [Y{P(SiMe_3_)_2_}_2_{μ-P(SiMe_3_)_2_}]_2_ (δ_P_: −104.8 ppm, ^1^*J*_YP_ = 122.4 Hz; −107.8 ppm, ^1^*J*_YP_ = 56.7 Hz),^52^ and the ^1^*J*_YbP_ coupling constant is larger than that observed for *trans*-[Yb(PPh_2_)_2_(THF)_4_] (δ_P_: −3.0 ppm, ^1^*J*_YbP_ = 840 Hz),^26^ [Yb{P(SiMe_3_)_2_}_2_(py)_4_] (δ_P_: −253.93 ppm, ^1^*J*_YbP_ = 925 Hz),^24^ and [Yb{P(SiMe_3_)_2_}_2_(18-crown-6)] (δ_P_: −265.58 ppm, ^1^*J*_YbP_ = 977 Hz).^24^ The ^171^Yb{^1^H} NMR spectrum of **1-Yb** shows the expected triplet resonance at δ_Yb_ = 682 ppm, relative to the [Yb(Cp*)_2_(THF)_2_] (Cp* = C_5_Me_5_) external reference, from the respective coupling of ^171^Yb nuclei with two equivalent 100% abundant *I* = 1/2 ^31^P nuclei (Figure 2b); this is comparable to the doublet of doublets resonance observed for the tetrameric Yb(II) phosphide complex [Yb{P[CH(SiMe_3_)_2_](C_6_H_4_-2-O)}(THF)]_4_ at δ_Yb_: 663.6 ppm (^1^*J*_YbP_ = 603 and 767 Hz),^51^ and the monomeric Yb(II) phosphide complexes [Yb{P(SiMe_3_)_2_}_2_(py)_4_] (δ_Yb_: 1070 ppm, ^1^*J*_YbP_ = 927 Hz)^24^ and [Yb{P(SiMe_3_)_2_}_2_(18-crown-6)] (δ_P_: 175 ppm, ^1^*J*_YbP_ = 977 Hz).^24^

### UV–Vis–NIR Spectroscopy

**Figure 3:**
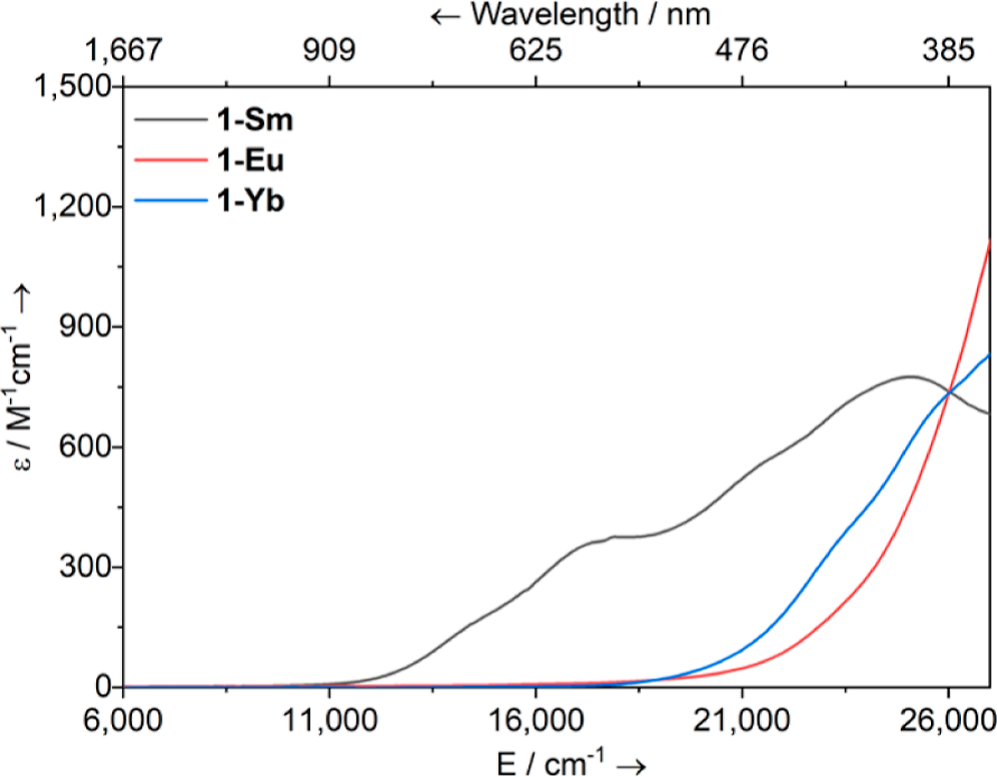
Overlaid electronic UV–vis–NIR absorption spectra of **1-Ln** (Ln = Sm, Eu and Yb) in toluene (2 mM) between 6000 and 27,000 cm^–1^ (1667–370 nm).

The electronic UV–vis–NIR absorption spectra of **1-Ln** were recorded as 2 mM toluene solutions (see Figure 3 for compiled spectra; individual spectra are available in Supporting Information Figures S22–S24). Notably, the absorption bands of Ln(II) complexes are highly sensitive to small changes in the local coordination environment. Strong broad absorptions are often seen in the visible region for Ln(II) complexes due to spin-allowed f–d transitions, where the stabilization of 5d orbitals compared to Ln(III) complexes lowers the energy of these Laporte-allowed transitions into the visible region to often mask weaker Laporte-forbidden f–f transitions.^53,54^ This is the case for complexes containing Sm(II) ions,^1^ and as such, toluene solutions of **1-Sm** are deep green, with two broad and intense absorption bands spread across the visible region with λ_max_ = 25,000 cm^–^1 (ε = 776 M^–1^ cm^–1^) and 17,182 cm^–1^ (ε = 355 M^–1^ cm^–1^). These absorptions are more intense than the feature in the UV region, which is typically assigned to charge transfer or intraligand absorptions.^1^ The spectrum of **1-Sm** is comparable with previously reported Sm(II) complexes such as [Sm{N(SiMe_3_)_2_}_2_]_2_ (λ_max_ = 28,600, 24,000, 18,000 and 16,200 cm^–1^)^55^ and [Sm{N(Si^*i*^Pr_3_)_2_}_2_] (λ_max_ = 29,900, 23,500 and 17,200 cm^–1^)^22^ as well as for [{Sm[P(SiMe_3_)_2_]_3_(THF)}_2_(μ-I)K_3_(THF)] (λ_max_ = 22,730 cm^–1^ (ε = 1575 M^–1^ cm^–1^), [Sm{P(SiMe_3_)_2_}_2_(py)_4_] (λ_max_ = 22,525 cm^–1^ (ε = 833 M^–1^ cm^–1^) and λ_max_ = 13,950 cm^–1^ (ε = 278 M^–1^ cm^–1^) and [Sm{P(SiMe_3_)_2_}_2_(18-crown-6)] (λ_max_ = 23,150 cm^–1^ (ε = 730 M^–1^ cm^–1^), λ_max_ = 17,360 cm^–1^ (ε = 488 M^–1^ cm^–1^) and λ_max_ = 15,385 cm^–1^ (ε = 526 M^–1^ cm–^1^).^24^ By contrast, pale solutions are generally observed for Eu(II) complexes as the Laporte-allowed f–d transitions tend to occur outside the visible region, and the f–f transitions are also spin-forbidden.^1^ As such, pale yellow toluene solutions of **1-Eu** are featureless apart from a band that tails into the visible region from the UV. Finally, for the Yb(II) complexes, no f–f transitions are possible as a result of the 4f^14^ closed shell electronic structure, but the crystal field (CF) imposed can influence colors strongly by varying the energies of the f–d transitions.^1^ Toluene solutions of **1-Yb** are pale yellow with a weak band tailing from the UV to the visible region, similar to **1-Eu**, but this band has two distinct shoulders at λ_max_ = 25,641 cm^–1^ (ε = 695 M^–1^ cm^–1^) and λ_max_ = 23,256 cm^–1^ (ε = 356 M^–1^ cm^–1^). UV–vis–NIR absorption spectroscopic data for Yb(II) phosphide complexes are scarce, but [Yb{(μ-P^*t*^Bu_2_)_2_Li(THF)}_2_] possesses absorption features at λ_max_ = 45,000, 37,000, 33,300 and 27,500 cm^–1^,^33^ with no bands reported at lower energies whereas [Yb{P(SiMe_3_)_2_}_2_(py)_4_] has a band at λ_max_ = 17,200 cm^–1^ (ε = 416 M^–1^ cm^–1^) and [Yb{P(SiMe_3_)_2_}_2_(18-crown-6)] has a band at λ_max_ = 24,750 cm^–1^ (ε = 720 M^–1^ cm^–1^).^24^

### Photoluminescence Studies

**Figure 4:**
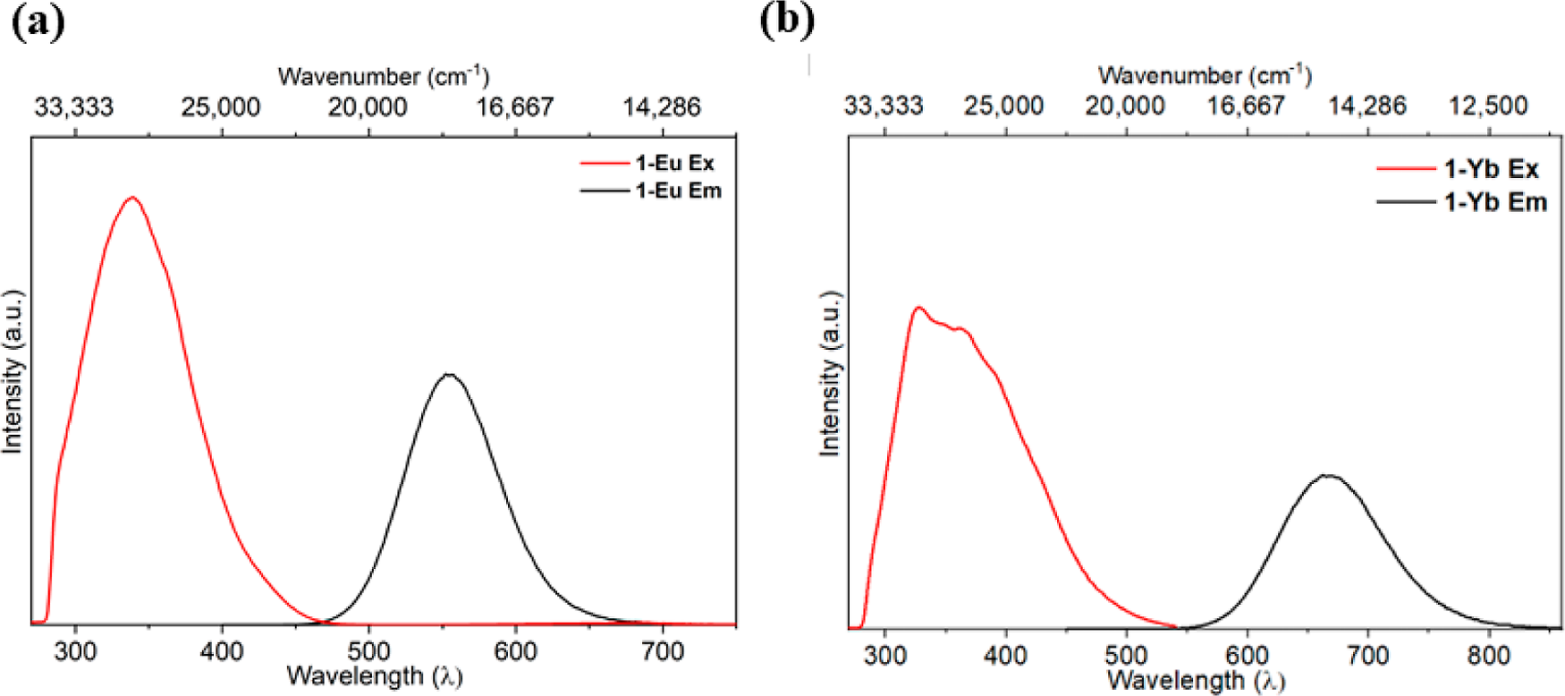
(a) Emission spectrum (**Em**), black trace and excitation spectrum (**Ex**), red trace of complex **1-Eu** in toluene (2.01 mM), room temperature. Excited at 300 nm (Ex), observed at 555 nm (Em). (b) Emission spectrum (**Em**), black trace and excitation spectrum (**Ex**), red trace of complex **1-Yb** in toluene (0.018 mM), room temperature. Excited at 300 nm (Ex), observed at 666 nm (Em).

We next examined the solution state luminescence properties of **1-Eu** and **1-Yb** at room temperature (Table 3, Figure 4 and Supporting Information Figures S25–S26). Since **1-Sm** possesses intense absorption bands that span the entire visible region, any emission arising from f–d or charge transfer electronic transitions is fully quenched by nonradiative decay mechanisms; hence, **1-Sm** is not included in this study. The luminescence of Ln(III) complexes is largely independent of the ligand field due to the “core-like” nature of the 4f-electrons, and these processes are well-understood.^56^ However, for Eu(II) and Yb(II), f–d excitations are possible that result in broader, shorter lived visible emission, observable since stabilization of the d-orbitals can change the relaxation pathways that are available to Ln(II) ions.^57,58^

**Table 3:** Summary of λ_ex_ (nm), λ_em_ (nm), and τ (ns) for Complexes **1-Eu** and **1-Yb**

complex	λ_ex_ (nm)	λ_em_ (nm)	τ_1_ (ns)
**1-Eu**	375	555	1424
**1-Yb**	375	666	

In **1-Eu** and **1-Yb** in a fluid toluene solution, excitation between 300 and 410 nm (Figure 4) results in intense vibrationally broadened emission in the green (λ_em_ = 555 nm) and red (λ_em_ = 666 nm) regions of the electromagnetic spectrum, respectively. These emissions are comparable to those seen for [K(2.2.2.cryptand)][Ln{N(Si^*t*^BuMe_2_)_2_}_3_],^59^ which exhibited emissions within the green (Ln = Eu, λ_em_ = 540 nm) and red (Ln = Yb, λ_em_ = 650 nm) regions, respectively, as well as for the emission reported for [Eu{N(Si^*i*^Pr_3_)_2_}_2_] (λ_em_ = 590 nm).^59^ The emission wavelengths are similar to those reported for [{Eu[P(SiMe_3_)_2_]_3_(THF)}_2_(μ-I)K_3_(THF)] (λ_em_ = 579 nm), *trans*-[Eu{P(SiMe_3_)_2_}_2_(py)_4_] (λ_em_ = 579 nm), and [Ln{P(SiMe_3_)_2_}_2_(18-crown-6)] (Ln = Eu, λ_em_ = 475 nm; Yb, λ_em_ = 475 nm).^24^ The emission bands in **1-Eu** and **1-Yb** are independent of the excitation wavelength in both cases, confirming that the emission originates from a common excited state. The excitation spectrum recorded at the emission maxima for **1-Eu** (Figure 4a) reveals a single, symmetric, broad excitation band centered at 340 nm with no contribution from higher energy charge transfer states. By contrast, the excitation spectrum of **1-Yb** (Figure 4b) possesses two features at 328 and 364 nm, again with no contributions from higher energy electronic excitations. The features in **1-Eu** and **1-Yb** are comparable to those seen for [K(2.2.2.cryptand)][Ln{N(Si^*t*^BuMe_2_)_2_}_3_(THF)_*n*_] (Ln = Eu, 280 and 330 nm; Yb, 320 and 360 nm).^59^ Together, these data indicate that the emission may be a result of deactivation of an f–d excited state that is initially populated by a higher energy charge transfer state.^60^

The luminescence lifetime of **1-Eu** at the emission maxima of 555 nm at room temperature was determined to be monoexponential at 1.42 μs (following excitation with a 375 nm picosecond pulsed diode laser, see Supporting Information Figure S26). This lifetime is slightly shorter than that recorded for [K(2.2.2.cryptand)][Eu{N(Si^*t*^BuMe_2_)_2_}_3_] (5.8 ± 0.005 μs)^59^ but is significantly longer than the luminescence lifetime observed for macrocyclic Eu(II) compounds in solution at room temperature,^61^ for example [Eu(benzo-15-crown-5)_2_][ClO_4_]_2_ (0.14 μs in MeOH)^62^ and [Eu(benzo-18-crown-6)_2_][Cl]_2_ (0.028 μs),^63^ and the recently reported phosphide complexes [{Eu[P(SiMe_3_)_2_]_3_(THF)}_2_(μ-I)K_3_(THF)] (τ_1_ = 811 ns, τ_2_ = 1760), *trans*-[Eu{P(SiMe_3_)_2_}_2_(py)_4_] (τ_1_ = 42 ns, τ_2_ = 771), and [Ln{P(SiMe_3_)_2_}_2_(18-crown-6)] (Ln = Eu, τ_1_ = 447 ns; Yb, τ_1_ = 16 ns, τ_2_ = 348).^24^ However, the lifetime of **1-Eu** is much shorter than for the near-linear Eu(II) complex [Eu{N(Si^*i*^Pr_3_)_2_}_2_] (49.6 ± 1.3 μs).^59^ By contrast, the luminescence lifetime of **1-Yb** could not be recorded due to it being shorter than the instrument response function (<0.5 ns), likely as a result of increased competitive nonradiative quenching processes in line with the energy gap law.^64^ As the luminescence properties of **1-Eu** and **1-Yb** were not remarkable, we did not perform dedicated low-temperature studies to determine quantum yields, which should be lower than 10%.

### Magnetism

**Figure 5:**
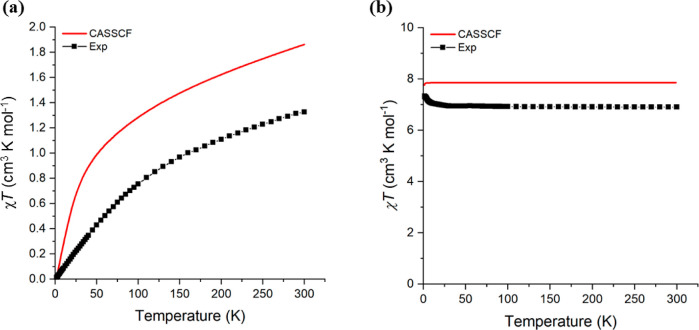
Magnetic susceptibility plots of (a) **1-Sm** and (b) **1-Eu**. Solid lines show CASSCF results.

The effective magnetic moment (μ_eff_) and molar magnetic susceptibility (χ_M_*T*) of powdered samples of paramagnetic **1-Sm** and **1-Eu** suspended in eicosane were examined by variable-temperature DC SQUID magnetometry and complete active space self-consistent field spin–orbit (CASSCF-SO) calculations (selected parameters compiled in Table 4; see Figure 5 and Supporting Information Figures S27–S30 for all magnetic data). There is reasonable agreement between measured and calculated magnetization values and the respective expected values for the free Sm(II) ion (4f^67^F_0_) for **1-Sm** and the free Eu(II) ion (4f^78^S_7/2_) for **1-Eu**. The 0.53 cm^3^ mol^–1^ K discrepancy between measured and CASSCF-predicted χ_M_*T* values at 300 K for **1-Sm** can be attributed to mixing of low-lying ^6^F excited states with the magnetic ground state. The saturation magnetization value for **1-Sm** is also lower than calculated, which indicates that the ground state is purer than the 80% *m*_J_ = 0, which is predicted by CASSCF, previously seen in [Sm(Cp^tt^)_3_] (Cp^tt^ = C_5_H_3_^*t*^Bu_2_-1,3).^65^ For **1-Eu**, the measured χ_M_*T* values are consistently lower than both the expected free ion and CASSCF-predicted values, with the measured χ_M_*T* 0.95 cm^3^ mol^–1^ K lower than the calculated value at 300 K. The absolute values of magnetization are also overpredicted for **1-Eu** by CASSCF. However, the χ*T* values are relatively unaffected by a decrease in temperature, indicative of a spin-only system and consistent with the presence of *S* = 7/2 Eu(II) ions. We therefore attribute discrepancies in the predicted and measured magnetic data for **1-Eu** to weighing errors and used EPR spectroscopy to investigate the electronic structure of this complex further.

**Table 4:** Product of the Molar Susceptibility and Temperature, χ_M_*T* (cm^3^ mol^–1^ K), of **1-Sm** and **1-Eu**, at 2 and 300 K as Determined by SQUID Magnetometry on Powder Samples, CASSCF Calculations, and Free Ion Values for Monomeric Ions

complex	SQUID magnetometry		CASSCF calculations		free ion^1^
	1.8 K	300 K	2 K	300 K	χ_M_*T*
**1-Sm**	0.01	1.33	0.31	1.86	1.45
**1-Eu**	7.30	6.91	7.83	7.86	7.88

### EPR Spectroscopy

The electronic structures of the Kramers ion complex **1-Eu** were investigated further by c.w. X-band (ca. 9.4 GHz) and Q-band (ca. 34 GHz) EPR spectroscopy.

**Figure 6:**
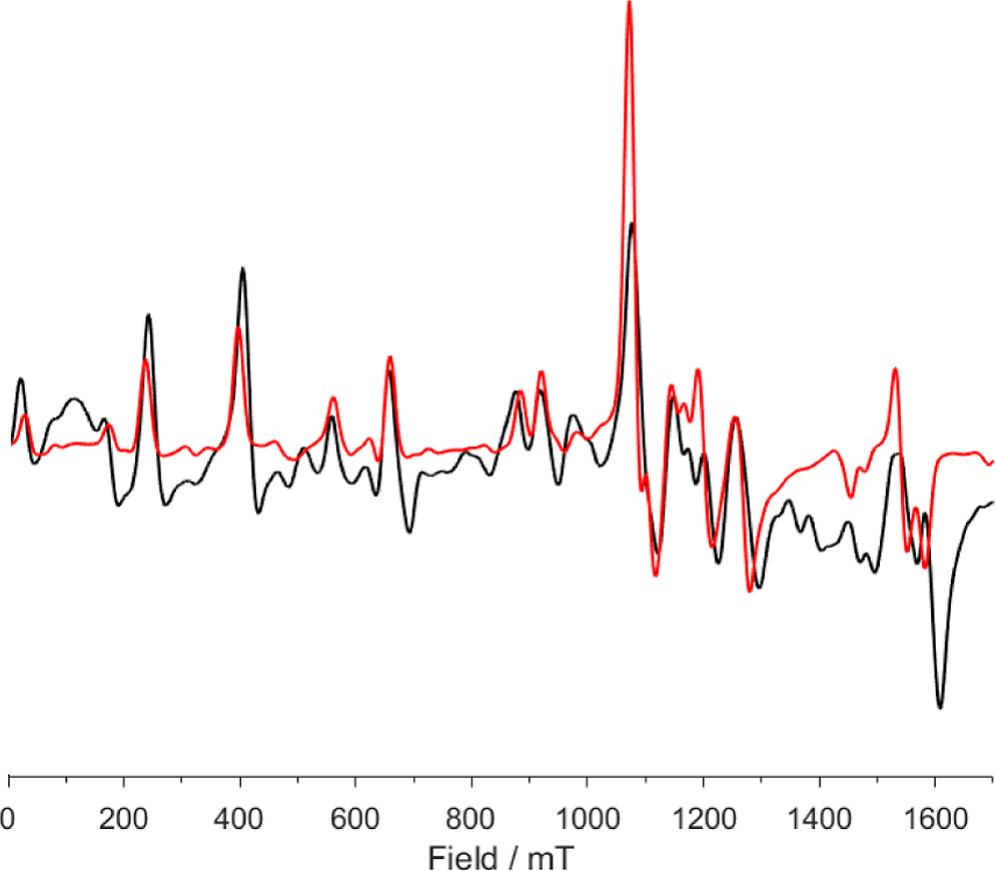
Experimental (black trace) and simulated (red trace) Q-band powder EPR spectra for **1-Eu** at 5 K.

Spectra from the solid state are well-resolved (Figure 6 and Supporting Information Figures S31–S39) with transitions across the entire magnetic field range measured (0–1.7 T); those from frozen solution (9:1, toluene/hexane, Supporting Information Figures S40–S48) are consistent with the solid-state spectra but are less well resolved presumably due to strain effects on the structure hence spin Hamiltonian parameters. Hence, we focus our discussion on solid-state spectra. The spectra were simulated, using EasySpin,^66^ with the simple Zeeman and zero-field splitting (ZFS) spin Hamiltonian
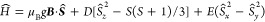
where *S* is the electron spin quantum number, μ_B_ is the Bohr magneton, ***B*** is the applied magnetic field, *g* is the electronic *g*-value (treated as isotropic), and *D* and *E* are the axial and rhombic components, respectively, of the ZFS interaction matrix. Higher-order ZFS terms, which are possible for *S* = 7/2, were neglected. Good simulations were found with *g* = 2.0, |*D*| = 4.8 GHz, and |*E*/*D*| = 0.25 (Figures 6 and S31). (Variation of *g* from 2.0, or making it significantly anisotropic, did not lead to improved agreement.) The high degree of rhombicity (the rhombic limit is given by |*E*/*D*| = 1/3) is consistent with the high distortion of the structure of **1-Eu** from trigonal bipyramidal. This rhombicity parameter is larger than we found for the related 6- and 8-coordinate complexes with terminal and *trans*-axial phosphides, [Ln{P(SiMe_3_)_2_}_2_(py)_4_] (*D* = −3.3 GHz, |*E*/*D*| = 0.19) and [Ln{P(SiMe_3_)_2_}_2_(18-crown-6)] (*D* = −5.4 GHz, |*E*/*D*| = 0.06), respectively.^24^ The magnitude of *D* for **1-Eu** is intermediate between those two complexes, and there does not appear to be an obvious correlation with Eu–P distances or P–Eu–P angles.

### DFT Calculations

The electron density of **1-Yb** was calculated using restricted Kohn–Sham density functional theory with the PBE^67^ functional. The Wiberg bond order (WBO) and delocalization index, δ(Yb–P), are both used to calculate the number of electrons shared between the nuclei of interest. For **1-Yb**, the average between the two Yb–P interactions results in a δ(Yb–P) = 0.25 and a WBO of 0.29. The average value of the electron density at the bond critical point for the Yb–P interactions, ρ_BCP_(Yb–P), is 0.027 au, which confirms that these interactions are weak. This is also corroborated with the highest occupied molecular orbital (MOs), which has a lack of orbital overlap between metal and ligand (see Supporting Information Figure S50).

### Ab Initio Calculations

The electronic structures of **1-Sm** and **1-Eu** were investigated by CASSCF-SO calculations using the OpenMolcas package (see Supporting Information Tables S6 and S7 and Figure S49).^68^ There is a mixed ground state for **1-Eu** consisting of |±1/2⟩ (41%), |±3/2⟩ (39%), |∓5/2⟩ (11%), and |± 7/2⟩ (6%). This ground Kramers doublet has effective *g*-values of *g*_1_ = 12.25, *g*_2_ = 2.81, and *g*_3_ = 1.24, where the largest *g*-value is approximately perpendicular with the P–Eu–P axis, suggesting that the intermediate *m*_J_ states are stabilized by both the hard O-donor atoms of THF and the two softer silylphosphide ligands. Conversely, **1-Sm** has a reasonably isolated nondegenerate ground state consisting of |0⟩ (80%).

## Conclusions

The solvated Ln(II) bis(triisopropylsilyl)phosphide complexes **1-Ln** (Ln = Sm, Eu, Yb) are straightforwardly prepared by salt metathesis methods and represent the first examples of structurally authenticated f-block complexes containing this bulky phosphide ligand. We find complementary and contrasting behavior of the solid-state structures and spectroscopic data of **1-Ln** to related Ln(II) complexes of the bulky bis(silyl)amide {N(Si^*i*^Pr_3_)_2_} and the smaller bis(silyl)phosphide {P(SiMe_3_)_2_}, driven by a combination of differing steric effects and ligand hardness. The reactivity of **1-Ln** and related derivatives should be rich due to the tendency of Ln(II) complexes to reduce substrates by single electron transfer;^1^ thus their redox chemistry will be investigated in future studies.

## Experimental Section

### General Caution!

PNa_3_ and early metal salts of {P(Si^*i*^Pr_3_)_2_}]_n_ are extremely pyrophoric and malodorous. It is therefore imperative that the anaerobic techniques outlined below are strictly followed and that these manipulations are performed on the smallest practicable scales; PNa_3_ was not isolated and was used in situ to mitigate risk. We recommend that readers familiarize themselves with the hazards and techniques outlined for the large-scale synthesis of P(SiMe_3_)_3_ (CAS: 15573-38-3) from red phosphorus, sodium metal, and chlorotrimethylsilane^41^ before proceeding with the synthesis of [Na{P(Si^*i*^Pr_3_)_2_]_n_^42^ and treat the procedures reported herein with the same level of caution as P(SiMe_3_)_3_ and its alkali metal salts. Prior to disposal as a nonhalogenated waste, toluene suspensions of all early metal {P(Si^*i*^Pr_3_)_2_}]_*n*_ salts were passivated by dropwise addition of 5:95 isopropanol/toluene solutions, followed by oxidation to P(V) products by dropwise addition of dilute solutions of iodine in ethanol. All manipulations were conducted under argon with the strict exclusion of oxygen and water by using Schlenk line and glovebox techniques. [LnI_2_(THF)_2_] (Ln = Sm, Eu, Yb) were prepared following literature procedures,^69^ while [Na{P(Si^*i*^Pr_3_)_2_}]_n_ was either prepared by the method below or by an improved synthesis.^41^ THF, pentanes, and toluene were purged with nitrogen and passed through columns containing the alumina catalyst and molecular sieves before being degassed, refilled with argon, and stored over activated 4 Å molecular sieves (THF) or a potassium mirror (pentanes, toluene) before use. For NMR spectroscopy, *d*_6_-benzene and *d*_8_-THF were dried by refluxing over K, and were vacuum transferred and degassed by three freeze–pump–thaw cycles before use. NMR spectra were recorded on a JEOL JNM-ECZ 400 MHz spectrometer operating at 399.78 (^1^H), 100.52 (^13^C{^1^H}), and 79.42 (^29^Si DEPT90) MHz, referenced to SiMe_4_, 161.83 (^31^P{^1^H}) MHz referenced to 85% H_3_PO_4_ and 70.67 (^171^Yb{^1^H}) MHz, referenced to [Yb(Cp*)_2_(THF)_2_]. FTIR spectra were recorded on microcrystalline solids using a Bruker Alpha spectrometer with a Platinum-ATR module. UV–vis–NIR spectroscopy were recorded on a PerkinElmer LAMBDA 750 spectrometer on 2 mM toluene solutions in a 1 cm path length cuvette and were corrected to a toluene reference cell. Elemental analysis was carried out by Mr Martin Jennings and Mrs Anne Davies at the Microanalytical service, School of Chemistry, the University of Manchester using a Thermo Scientific Flash Smart Elemental Analyzer with D4001 Flat Base Smooth Wall Tin Capsules (6 × 3 mm). Elemental analysis results for **1-Ln** reproducibly gave low carbon values; this has consistently been seen for {N(Si^*i*^Pr_3_)_2_} complexes and we have previously attributed this observation to the formation of carbides from incomplete combustion.^21,22,42−45^

The crystal data for [Ln{P(Si^*i*^Pr_3_)_2_}_2_(THF)_*x*_] (**1-Ln**; Ln = Sm, Eu, *x* = 3; Ln = Yb, *x* = 2) and [Na{P(Si^*i*^Pr_3_)_2_}(DME)_2_] are compiled in Tables S1 and S2. Crystals were examined using a Rigaku XtalLAB AFC11 diffractometer equipped with a CCD area detector and graphite-monochromated Cu Kα (λ = 1.54178 Å) or Mo Kα radiation (λ = 0.71073 Å). Intensities were integrated from data recorded on 1° frames by ω rotation. Cell parameters were refined from the observed positions of all of the strong reflections in each data set. A Gaussian grid face-indexed correction was used to account for X-ray absorption.^70^ The structures were solved using SHELXS.^71^ Data sets were refined by full-matrix least-squares on all unique *F*^2^ values,^72^ with anisotropic displacement parameters for all non-hydrogen atoms, and with constrained riding hydrogen geometries; *U*_iso_(H) was set at 1.2 (1.5 for methyl groups) times *U*_eq_ of the parent atom. The largest features in final difference syntheses were close to those of heavy atoms and were of no chemical significance. CrysAlisPro^70^ was used for control and integration, and SHELX^71,72^ was employed through OLEX2^73^ for structure solution and refinement. ORTEP-3^74^ and POV-Ray^75^ were employed for molecular graphics.

Steady-state and time-resolved emission spectra were recorded at room temperature from 300 to 800 nm in Youngs tap-appended 10 mm path length quartz cuvettes on an Edinburgh Instruments FLS-1000 photoluminescence spectrometer equipped with a 450 W steady state xenon lamp, a 60 W microsecond pulsed xenon flash lamp, and interchangeable picosecond pulsed diode lasers (EPL-375 and EPL-405), with single 325 mm focal length excitation and emission monochromators in Czerny Turner configuration and a red sensitive photomultiplier in Peltier (air cooled) 53 housing (Hamamatsu R928P). Lifetime data were recorded following excitation at 375 nm by the EPL-375 diode laser using time-correlated single photon counting. Lifetimes were obtained by a tail fit on the data obtained. Plotting, fitting, and analysis of data were carried out using the built-in instrumental software and Origin 2019b. All data were fitted with exponential decay models and the goodness of fit evaluated by minimization of the residuals squared, χ^2^ and *R*^2^ analysis.

Magnetic data were collected on a Quantum Design MPMS3 superconducting quantum interference device (SQUID) magnetometer using doubly recrystallized powdered samples. Samples were prepared in an NMR tube containing finely ground powders of crystalline material (**1-Sm**: 32.3 mg; **1-Eu**: 15.2 mg). Eicosane was added (**1-Sm**: 17.5 mg; **1-Eu**: 7.2 mg) and the samples briefly heated to melt this wax, which then acted as a restraint when the samples were allowed to cool and set; these tubes were then flame-sealed under vacuum to ca. 5 cm in length. The ampules were mounted in plastic straws and held in place with diamagnetic tape. Samples were carefully checked for purity and data reproducibility among several independently prepared batches. Measurements were corrected for the contribution of the blank sample holders (flame-sealed Wilmad NMR tube and straw) and eicosane matrix, corrected for both the shapes of the sample using the MPMS3 Geometry Simulator (correction factors 0.974–1.075) and the diamagnetic contributions, which were approximated as the molecular weights multiplied by 0.5 × 10^–6^ cm^3^ K mol^–1^. Variable temperature magnetic susceptibility measurements were collected under an applied field (**1-Sm**: 0.1 T, **1-Eu**: 0.1 T) using VSM mode with 5 mm amplitude and 2 s averaging time. Isothermal magnetization measurements were performed in DC scan mode with a 40 mm scan length and 6 s scan time.

Continuous wave X-band (ca. 9.4 GHz) spectra were recorded with a Bruker EMX spectrometer fitted with a Super High Q X-band resonator and at Q-band (ca. 33.9 GHz) microwave frequency using a Bruker EMX300 spectrometer. Polycrystalline and frozen solution (9:1 toluene/hexane, 10 mM) samples of **1-Eu** were sealed in quartz X-band and Q-band EPR tubes in vacuo; samples were lightly ground with a mortar and pestle to reduce the amount of sample decomposition, but we note that some effects due to polycrystallinity remain in the spectra below. The presence of a very sharp resonance at *g* = 2.00 is attributed to an impurity in the quartz EPR tubes and serves as an internal reference for comparing relative intensities.

OpenMolcas^68^ (version v19.11-d14be45) was used to perform CASSCF-SO calculations on **1-Sm** and **1-Eu** to determine the electronic structure. The molecular geometry from a single crystal XRD structure was used with no optimization, selecting a single molecule from the asymmetric unit and taking the largest disorder component only. Electron integrals were calculated in the SEWARD module using basis sets from the ANO-RCC library^76,77^ with VTZP quality on the metal atom, VDZP quality on the P and O atoms, and VDZ quality on all other atoms, employing the second-order DKH Hamiltonian to account for scalar relativistic effects. Resolution of identity Cholesky decomposition of the two-electron integrals with on-the-fly auxiliary atomic compact Cholesky decomposition basis sets was used to save disk space and to reduce computational demand.^78^ The MOs were optimized using state-averaged CASSCF (SA-CASSCF) calculations in the RASSCF module with a CAS(*n*, 7) calculation where the active space included the valence 4f electrons (*n*) and the seven 4f orbitals. For **1-Sm**, SA-CASSCF calculations were performed considering the 7 lowest septets, and then all states were mixed via spin-orbit coupling. For **1-Eu**, SA-CASSCF calculations were performed for all possible spins and the following number of roots: 1 octet, 48 sextets, 392 quartets, and 560 doublets. A subset of these roots were then mixed by spin orbit coupling in the RASSI module, where 1 octet, 48 sextets, 119 quartets, and 113 doublets were included. SINGLE_ANISO was used to decompose the resulting spin–orbit wave functions into the CF Hamiltonian formalism.^79^ Diamond was employed for molecular graphics.^80^

Density functional theory was used to optimize the hydrogen positions of **1-Yb**, while all other atoms were kept frozen at the crystal structure positions; this was done using the PBE^67^ density functional with the Stuttgart RSC 1997 ECP for Yb^81^ and cc-pDVZ^82^ basis set for the ligand atoms, and dispersion interactions were included with the D3 dispersion correction.^83,84^ All DFT calculations were performed with Gaussian 16 Rev C.01.^84^ Multiwfn 3.8^85^ was used to perform an analysis of the total electron density.

### [Na{P(Si^*i*^Pr_3_)_2_}]

A three-neck 500 mL round-bottom Schlenk was charged with red phosphorus (3.100 g, 100.0 mmol) and dried under vacuum for ca. 12 h. DME (ca. 250 mL) and naphthalene (3.00 g, 23.4 mmol) were added. Chunks of Na washed in hexane (6.897 g, 300.0 mmol, 3 equiv) were added in small portions over the space of 30 min. The reaction mixture was stirred at reflux (100 °C) for 16 h and became dark green in color. A dropping funnel was charged with Si^*i*^Pr_3_Cl (45.0 mL, 2.1 equiv) and this was added dropwise to the reaction mixture at room temperature. The reaction mixture was stirred at reflux (100 °C) overnight and became purple in color. This was allowed to cool and was filtered into a 3-neck round-bottom Schlenk (500 mL) through a fritted Schlenk via a wide-bore cannula. The solvent was removed from the resultant yellow-orange solution under vacuum to yield a yellow/orange oily solid, with crystalline material forming when dried in vacuo. The oil was washed with pentane (100 mL), filtered and the remaining off-white solid was dried in vacuo. Yield = 5.489 g, 10 mmol, 10%. Crystals of [Na{P(Si^*i*^Pr_3_)_2_}(DME)_2_] were obtained by recrystallization of the off-white solid from a saturated DME solution, and this was found to desolvate under a vacuum. Anal. Calcd (%) for C_18_H_42_PSi_2_Na: C, 58.64; H, 11.48. Found (%): C, 58.29; H, 11.55. ^1^H NMR (400 MHz, *d*_8_-THF, 298 K): δ 1.02 (m, 6H, C*H*(CH_3_)_2_), 1.11 (d, ^3^*J*_HH_ = 6.9 Hz, 36H, CH(C*H*_3_)_2_). ^13^C{^1^H} NMR (101 MHz, *d*_8_-THF, 298 K): δ 17.47 (d, ^2^*J*_PC_ = 9.9 Hz, *C*H(CH_3_)_2_, 21.18 (d, ^3^*J*_PC_ = 3.8 Hz, CH(*C*H_3_)_2_). ^29^Si DEPT90 NMR (79 MHz, *d*_8_-THF, 298 K): δ 20.65 (d, ^1^*J*_PSi_ = 59.8 Hz (P*Si*). ^31^P{^1^H} NMR (162 MHz, *d*_8_-THF, 298 K): δ −384.26 (s, Na*P*). FTIR ν/cm^–1^: 2937 (s), 2859 (s), 1461 (m), 1359 (m), 1239 (w), 1015 (m), 1011 (s), 876 (s), 619 (s).

### [Sm{P(Si^*i*^Pr_3_)_2_}_2_(THF)_3_] (**1-Sm**)

A suspension of [SmI_2_(THF)_2_] (0.5490 g, 1.00 mmol) and [Na{P(Si^*i*^Pr_3_)_2_}]_*n*_ (1.1698 g, 2.00 mmol) in toluene (50 mL) was heated at 80 °C for 4 days to give a green-brown reaction mixture. The volatiles were removed in vacuo, and the product was extracted with pentanes (20 mL) and filtered. The filtrate was concentrated to ca. 5 mL, and a few drops of THF were added before the flask was stored at −30 °C to give dark green crystals of the title complex, which were isolated and dried in vacuo. Yield: 0.6817 g, 0.644 mmol, 64%. Anal. Calcd (%) for C_48_H_108_O_3_P_2_Si_4_Sm: C, 54.49; H, 10.29. Found (%): C, 52.66; H, 10.49. ^1^H NMR (400 MHz, *d*_6_-benzene, 298 K): δ −0.90 (br, 12H, THF–C*H*_2_), 1.15 (br, 12H, C*H*(CH_3_)_2_), 1.29 (br, 72H, CH(C*H*_3_)_2_), 5.68 (br, 12H, THF–C*H*_2_). ^13^C{^1^H} NMR (101 MHz, *d*_6_-benzene, 298 K): δ 24.98 (*C*H(CH_3_)_2_), 28.78 (CH(*C*H_3_)_2_), THF signals not observed. ^29^Si DEPT90 NMR (79 MHz, *d*_6_-benzene, 298 K): no signal was observed for **1-Sm** between +250 and −250 ppm due to paramagnetic broadening. ^31^P{^1^H} NMR (162 MHz, *d*_6_-benzene, 298 K): No signal observed for **1-Sm** between +1000 and −1000 ppm due to paramagnetic broadening. FTIR ν/cm^–1^: 2933 (m), 2853 (s), 1461 (m), 1375 (w), 1258 (w), 1071 (w), 1015 (m), 882 (s).

### [Eu{P(Si^*i*^Pr_3_)_2_}_2_(THF)_3_] (**1-Eu**)

The compound was prepared by the same method as for **1-Sm** using [EuI_2_(THF)_2_] (0.4275 g, 0.777 mmol) and [Na{P(Si^*i*^Pr_3_)_2_}]_*n*_ (0.8533 g, 1.555 mmol) to give yellow crystals of **1-Eu**. Yield = 0.3537 g, 0.334 mmol, 43%. Anal. Calcd (%) for C_48_H_108_O_3_P_2_Si_4_Eu: C, 54.41; H, 10.27. Found (%): C, 54.05; H, 10.22. ^1^H NMR (400 MHz, *d*_6_-benzene, 298 K): data between +200 and −200 ppm were not interpreted due to paramagnetic broadening. ^13^C{^1^H} NMR (101 MHz, *d*_6_-benzene, 298 K): not observed due to paramagnetic broadening. ^29^Si DEPT90 NMR (79 MHz, *d*_6_-benzene, 298 K): not observed due to paramagnetic broadening. ^31^P{^1^H} NMR (162 MHz, *d*_6_-benzene, 298 K): not observed due to paramagnetic broadening. FTIR ν/cm^–1^: 2941 (m), 2859 (s), 1463 (m), 1377 (w), 1225 (w), 1070 (w), 1007 (m), 989 (m), 873 (s).

### [Yb{P(Si^*i*^Pr_3_)_2_}_2_(THF)_2_] (**1-Yb**)

The compound was prepared by the same method as that for **1-Sm** using [YbI_2_(THF)_2_] (0.5711 g, 1 mmol) and [Na{P(Si^*i*^Pr_3_)_2_}]_*n*_ (1.1698 g, 2 mmol) to give yellow crystals of **1-Yb**. Yield = 0.4812 g, 0.477 mmol, 48%. Anal. Calcd (%) for C_44_H_100_O_2_P_2_Si_4_Yb: C, 52.40; H, 9.99. Found (%): C, 50.65; H, 10.24. ^1^H NMR (400 MHz, *d*_6_-benzene, 298 K): δ 1.41 (br, 92H, C*H*(C*H*_3_)_2_, C*H*(C*H*_3_)_2_ and THF–C*H*_2_), 3.85 (m, br, 8H, THF–C*H*_2_). ^13^C{^1^H} NMR (101 MHz, *d*_6_-benzene, 298 K): δ 16.90 (Vir.t, ^2^*J*_PC_ = 10.3 Hz, *C*H(CH_3_)_2_), 20.57 (Vir.t, ^3^*J*_PC_ = 3.6 Hz, CH(*C*H_3_)_2_), THF resonances not observed. ^29^Si DEPT90 NMR (79 MHz, *d*_6_-benzene, 298 K): δ 24.30, (Vir. t, ^1^*J*_PSi_ = 15.8 Hz, (P*Si*). ^31^P{^1^H} NMR (162 MHz, *d*_6_-benzene, 298 K): δ −301.10 (s, ^1^*J*_YbP_ = 1382.1 Hz, ^1^*J*_PSi_ = 18.1 Hz, Yb*P*). ^171^Yb{^1^H} NMR (71 MHz, *d*_6_-benzene, 298 K): δ 682.0 (t, ^1^*J*_YbP_ = 1382.9 Hz, YbP). FTIR ν/cm^–1^: 2947 (m), 2869 (s), 1469 (m), 1379 (w), 1235 (w), 1069 (w), 1011 (s), 873 (s).

## Data Availability

A preprint of this article was previously deposited on ChemRxiv.^86^ Research data files supporting this publication are available from FigShare at https://figshare.com/articles/dataset/Ln_II_Ptt_ResearchData/26236493?file=49605414.

## Supporting Information Available

Additional experimental details, materials, and methods, and data associated with this manuscript (PDF)
