# Supplementary material for: Synthesis and Characterization of Solvated Lanthanide(II) Bis(triisopropylsilyl)phosphide Complexes
Source: Inorg Chem. 2024 Oct 18;63(43):20295–306. doi: 10.1021/acs.inorgchem.4c03135 (PMC11523230; doi:10.1021/acs.inorgchem.4c03135)
Supplement: Supplementary file 1 — ic4c03135_si_001.pdf [file ic4c03135_si_001.pdf]

Supporting Information

Synthesis and characterization of solvated lanthanide(II) bis(triisopropylsilyl)phosphide complexes

Jack Baldwin, Adam Brookfield, George F. S. Whitehead, Louise S. Natrajan,* Eric J. L. McInnes,* Meagan S. Oakley,* David P. Mills*

Department of Chemistry, The University of Manchester, Oxford Road, Manchester, M13 9PL, UK.

*E-mail: louise.natrajan@manchester.ac.uk; eric.mcinnes@manchester.ac.uk; meagan.oakley@manchester.ac.uk; david.mills@manchester.ac.uk.

Contents

1. **ATR-IR Spectroscopy** S2
2. **Crystallographic Details** S5
3. **Molecular Structures** S8
4. **NMR Spectroscopy** S9
5. **UV-Vis-NIR Spectroscopy** S16
6. **Luminescence Properties: 1-Eu, 1-Yb** S18
7. **SQUID Magnetometry: 1-Sm, 1-Eu** S20
8. **EPR Spectroscopy: 1-Eu** S22
9. ***Ab initio* Calculations: 1-Eu** S32
10. **DFT Calculations: 1-Yb** S34

1. ATR-IR Spectroscopy

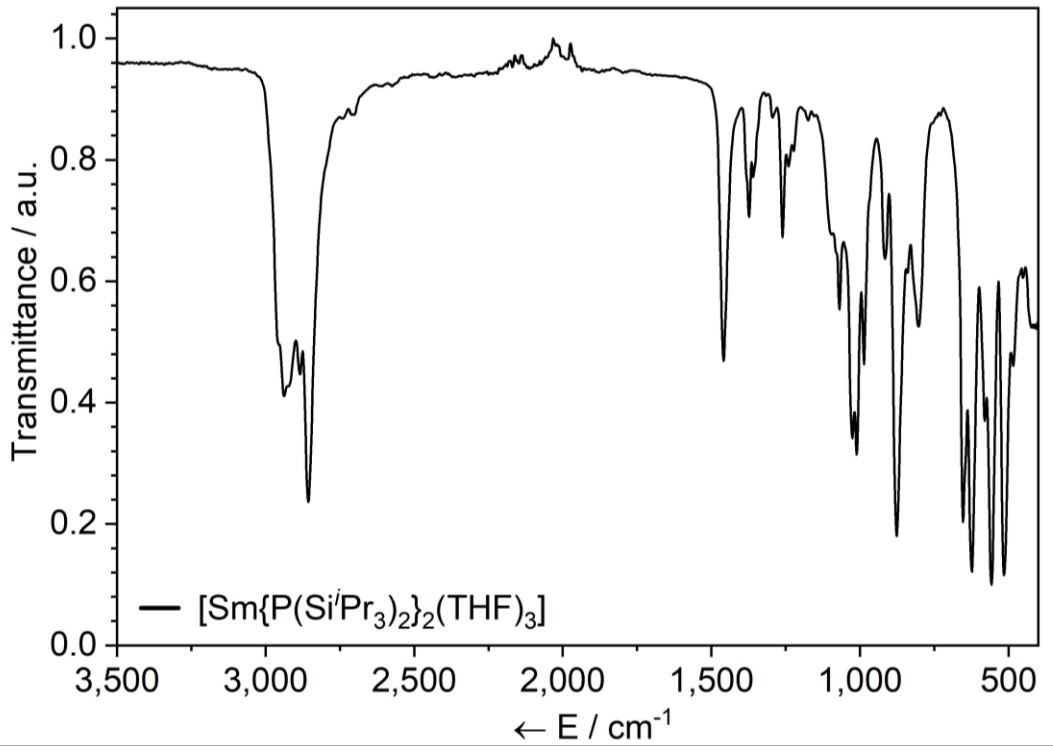

Figure S1. ATR-IR spectrum of **1-Sm** between 398-4000 cm^{-1}.

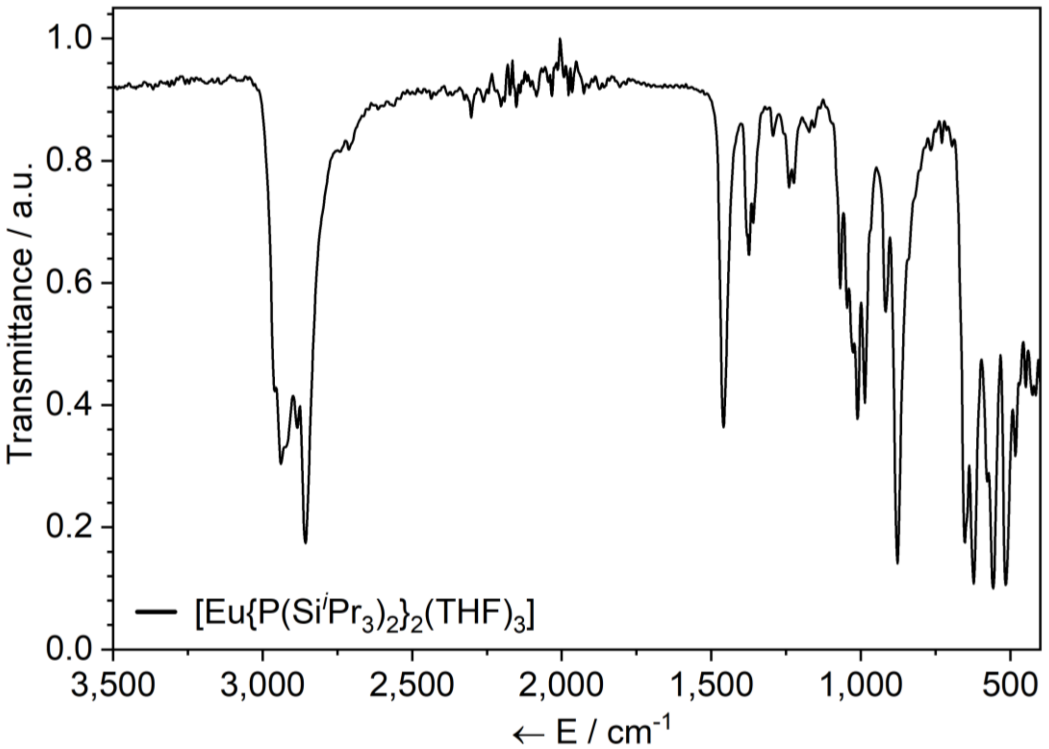

Figure S2. ATR-IR spectrum of **1-Eu** between 398-4000 cm^{-1}.

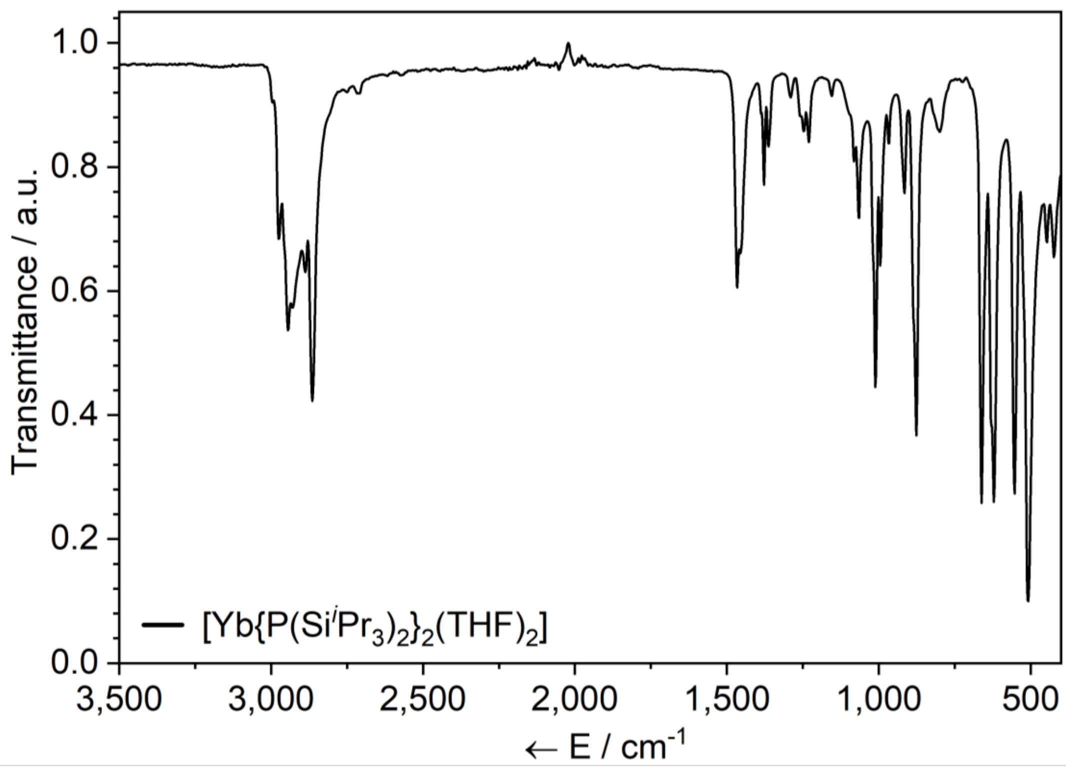

Figure S3. ATR-IR spectrum of **1-Yb** between 398-4000 cm^{-1}.

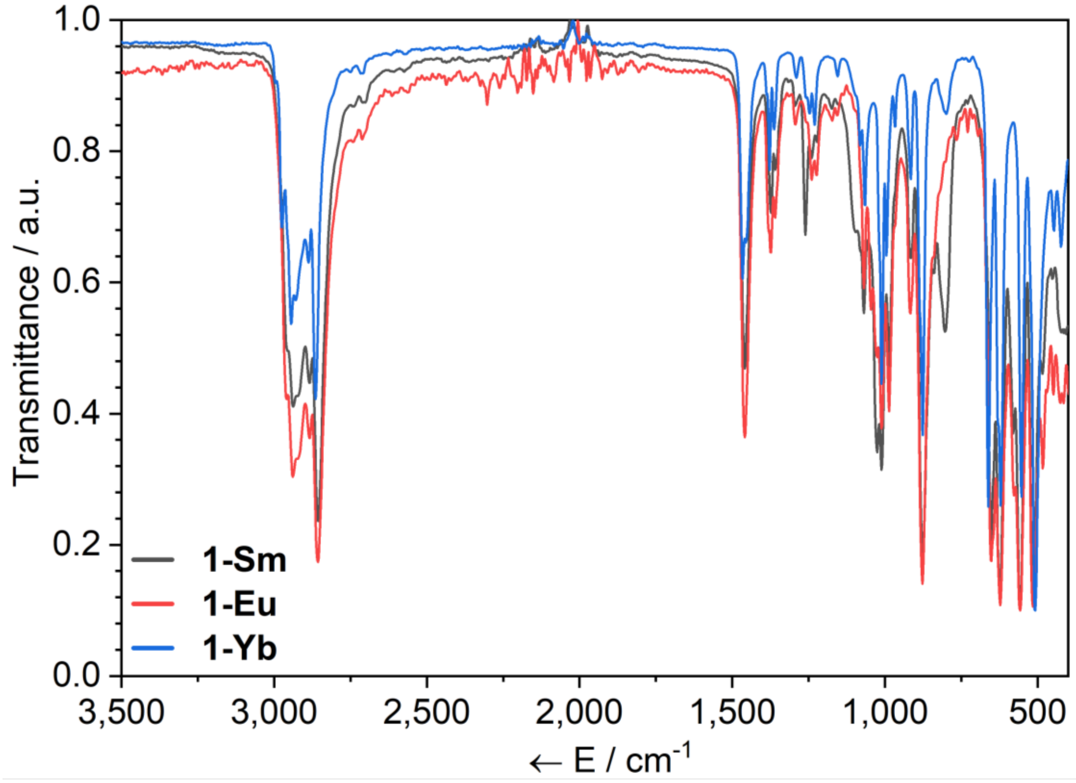

Figure S4. ATR-IR spectra of **1-Ln** between 398-4000 cm^{-1}.

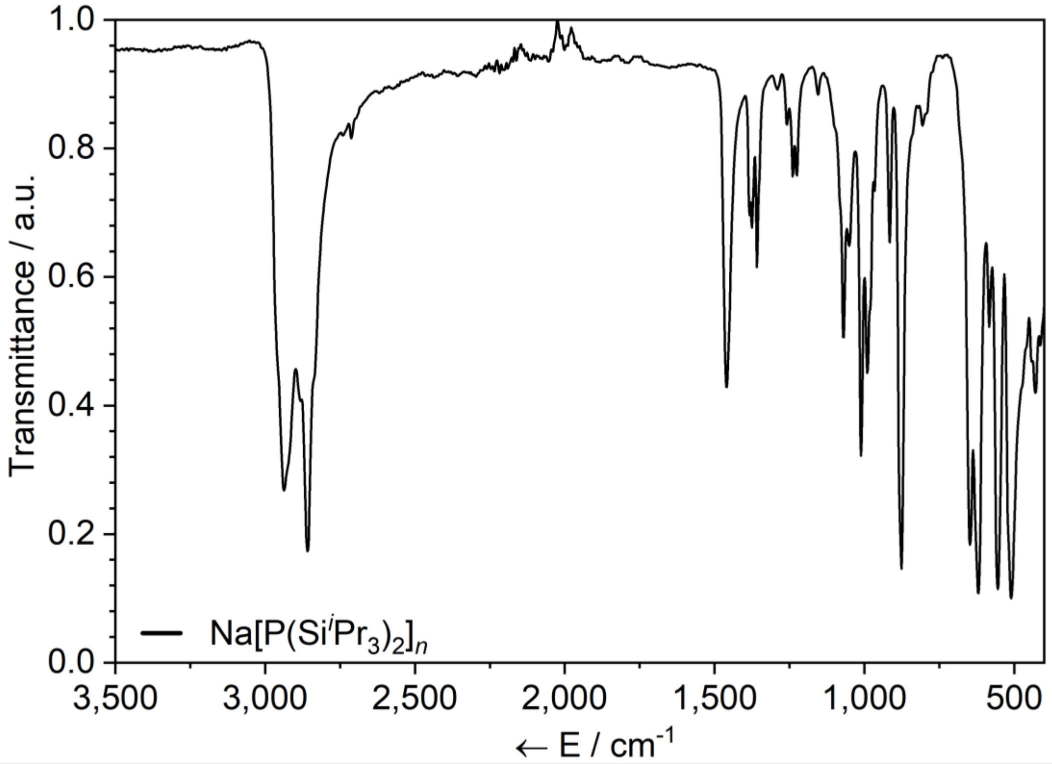

Figure S5. ATR-IR spectrum of [Na{P(Si^{i}Pr$_3$)$_2$}]$_n$ between 398-4000 cm^{-1}.

2. Crystallographic Details

Table S1. Crystallographic data for [Ln{P(Si^{i}Pr$_3$)$_2$}$_2$(THF)$_x$] (**1-Ln**; Ln = Sm, Eu, *x* = 3; Ln = Yb, *x* = 2). [a]Conventional $R = \Sigma||F_o| - |F_c||/\Sigma|F_o|$; $R_w = [\Sigma w(F_o^2 - F_c^2)^2/\Sigma w(F_o^2)^2]^{1/2}$; $S = [\Sigma w(F_o^2 - F_c^2)^2$ /no. data – no. params)]$^{1/2}$ for all data.

	**1-Sm**	**1-Eu**	**1-Yb**
Formula	$C_{48}H_{108}O_3P_2Si_4Sm$	$C_{48}H_{108}O_3P_2Si_4Eu$	$C_{44}H_{100}O_2P_2Si_4Yb$
Fw	1057.99	1059.60	1008.57
cryst size, mm	0.126×0.105×0.071	0.213×0.089×0.053	0.176×0.158×0.056
cryst syst	Monoclinic	Monoclinic	Monoclinic
space group	$P2_1/n$	$P2_1/n$	$P2_1/n$
a, Å	13.6676(3)	13.6566(2)	11.5006(2)
b, Å	23.7445(5)	23.6417(5)	28.3800(5)
c, Å	18.1844(4)	18.1535(3)	17.4152(4)
α, °	90	90	90
β, °	99.450(2)	99.4915(19)	105.922(2)
γ, °	90	90	90
V, Å^3	5821.3(2)	5780.92(19)	5466.03(19)
Z	4	4	4
ρ_{calc}, g/cm^3	1.207	1.218	1.226
μ, mm^{-1}	1.180	1.258	4.776
F(000)	2272	2276	2144
no. of unique reflns, *R*int	13310	13755	11862
R, *Rwa* ($F^2 > 2\sigma(F^2)$)	0.0403, 0.0671	0.0527, 0.1136	0.0294, 0.0707
S[a]	1.025	1.040	1.019
Rint	0.0336	0.0638	0.0592
max., min. diff map, e Å^{-3}	0.557, –0.515	2.309, –0.959	0.997, –0.852

Table S2. Crystallographic data for [Na{P(Si^{i}Pr$_3$)$_2$}(DME)$_2$]. ^{a}Conventional $R = \Sigma||F_o| - |F_c||/\Sigma|F_o|$; $R_w = [\Sigma w(F_o^2 - F_c^2)^2/\Sigma w(F_o^2)^2]^{1/2}$; $S = [\Sigma w(F_o^2 - F_c^2)^2$ /no. data – no. params$)]^{1/2}$ for all data.

	**[Na{P(Si^{i}Pr$_3$)$_2$}(DME)$_2$]**
Formula	$C_{26}H_{62}NaO_4PSi_2$
Fw	548.91
cryst size, mm	0.42×0.344×0.099
cryst syst	Orthorhombic
space group	$P_{ca}2_1$
a, Å	19.0767(9)
b, Å	19.3274(10)
c, Å	18.2815(9)
α, °	90
β, °	90
γ, °	90
V, Å^3	6740.5(6)
Z	4
ρ_{calc}, g/cm^3	1.082
μ, mm^{-1}	0.192
F(000)	2432
no. of unique reflns, *R*int	15026
R, *Rwa* ($F^2 > 2\sigma(F^2)$)	0.0515, 0.0842
S^a	1.015
Rint	0.0478
max., min. diff map, e Å^{-3}	0.321, –0.260

Table S3. Continuous Shape Measures (CShM) calculations for **1-Ln** (Ln = Sm, Eu).

Structure[a]	PP-5	vOC-5	TBPY-5	SPY-5	JTBPY-5
**1-Sm CShM**	32.232	3.728	4.285	5.467	8.547
**1-Eu CShM**	32.249	3.718	4.316	5.475	8.567

[a] PP-5 Pentagon (D_{5h})

vOC-5 Vacant octahedron (C_{4v})

TBPY-5 Trigonal bipyramid (D_{3h})

SPY-5 Spherical square pyramid (C_{4v})

JTBPY-5 Johnson trigonal bipyramid J12 (D_{3h})

Table S4. Continuous Shape Measures (CShM) calculations for **1-Yb**.

Structure[a]	SP-4	T-4	SS-4	vTBPY-4
**1-Yb CShM**	28.074	3.311	4.015	2.306

[a] SP-4 Square (D_{4h})

T-4 Tetrahedron (T_d)

SS-4 Seesaw (C_{2v})

vTBPY-4 Vacant trigonal bipyramid (C_{3v})

Table S5. τ_4 and τ_5 shape analysis for **1-M** (M = Sm, Eu, Yb).

Structure*	τ_4 [a]	τ_5 [b]
**1-Sm**	-	0.8535
**1-Eu**	-	0.8560
**1-Yb**	0.7976	-

$$^{a}\ \tau_4 = \frac{360° - (\alpha + \beta)}{360° - \theta} \approx -0.00709\alpha - 0.00709\beta + 2.55 \quad (1)$$

$$^{b}\ \tau_5 = \frac{\beta - \alpha}{60°} \approx -0.01667\alpha + 0.01667\beta \quad (2)$$

where: β > α are the two greatest valence angles of the coordination center.

* When τ_4 = 0, square planar, when τ_4 = 1, tetrahedral.

When τ_5 = 0, square pyramidal, when τ_5 = 1, trigonal bipyramidal.

3. Molecular Structures

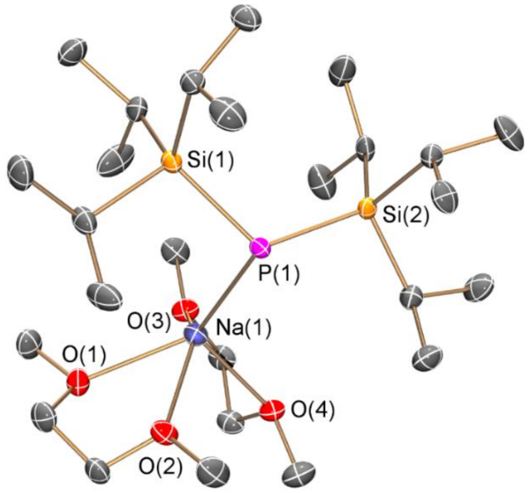

Figure S6. Solid state structure of [Na{P(Si^{i}Pr$_3$)$_2$}(DME)$_2$], with selected atomic labeling; Na = blue, P = magenta, Si = yellow, O = red, C = gray. Displacement ellipsoids set at 50% probability level, hydrogen atoms omitted for clarity.

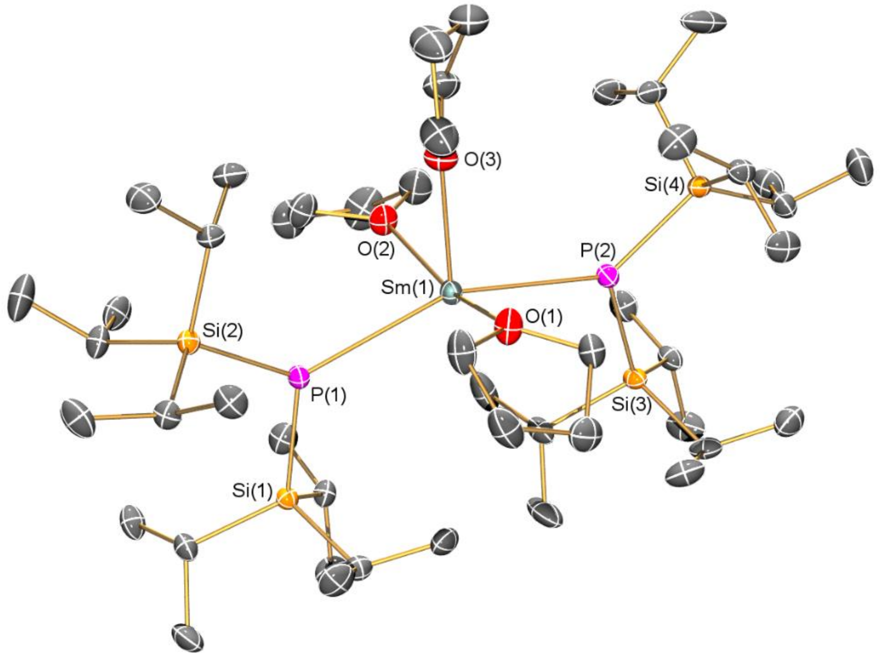

Figure S7. Solid state structure of **1-Sm**, with selected atomic labeling; Sm = cyan, P = magenta, Si = yellow, O = red, C = gray. Displacement ellipsoids set at 50% probability level, hydrogen atoms omitted for clarity.

4. NMR Spectroscopy

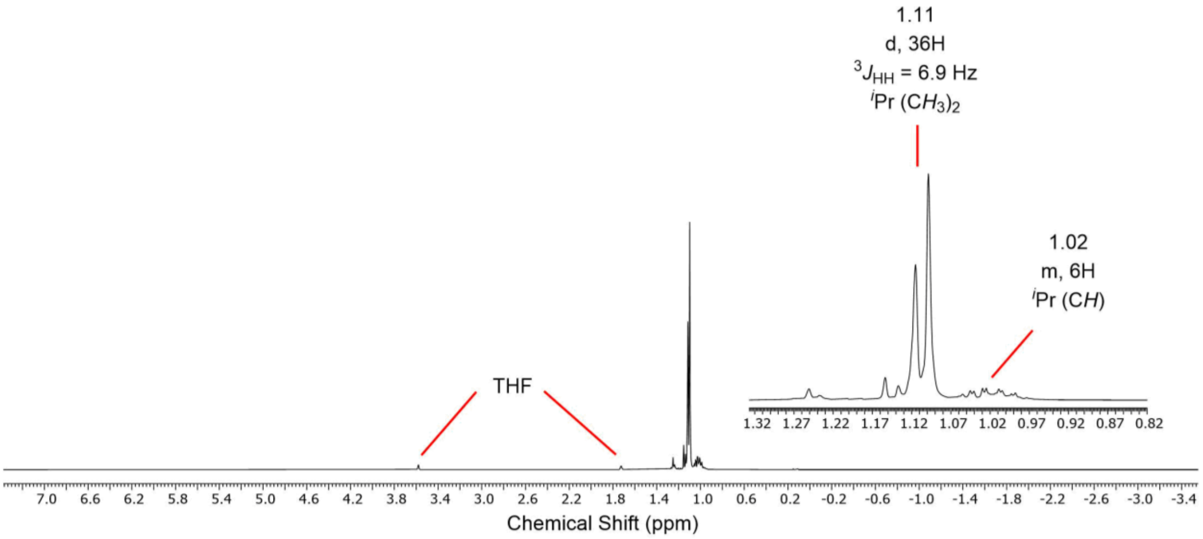

Figure S8. ^{1}H NMR spectrum (400 MHz) of [Na{P(Si^{i}Pr$_3$)$_2$}] in d_8-THF.

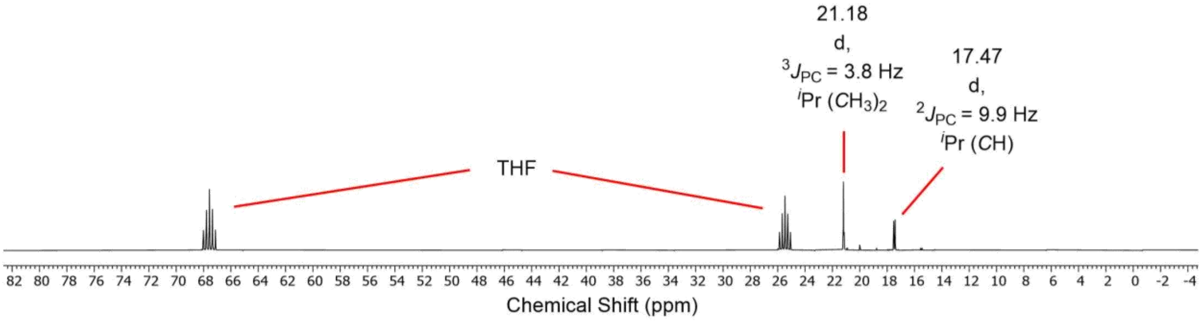

Figure S9. $^{13}C\{^{1}H\}$ NMR spectrum (101 MHz) of [Na{P(Si^{i}Pr$_3$)$_2$}] in d_8-THF.

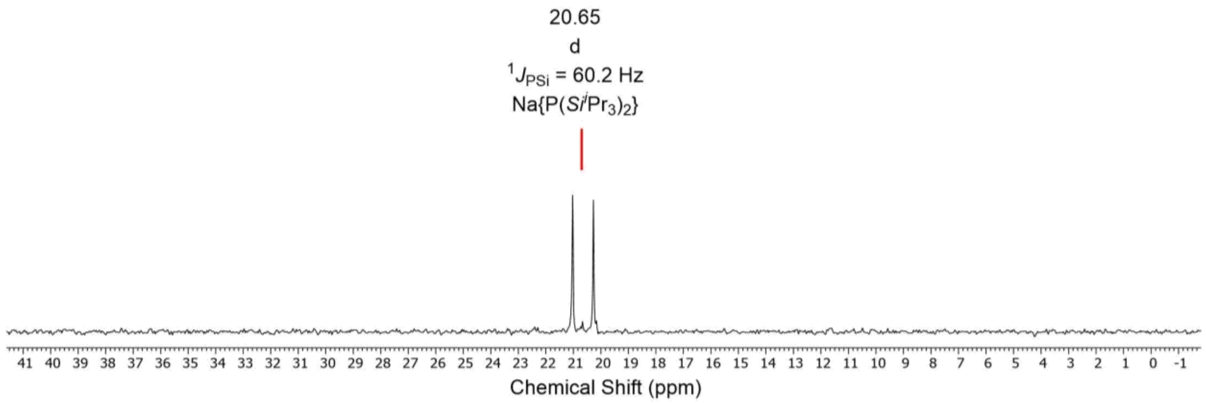

Figure S10. ^{29}Si DEPT90 NMR spectrum (79 MHz) of [Na{P(Si^{i}Pr$_3$)$_2$}] in d_8-THF.

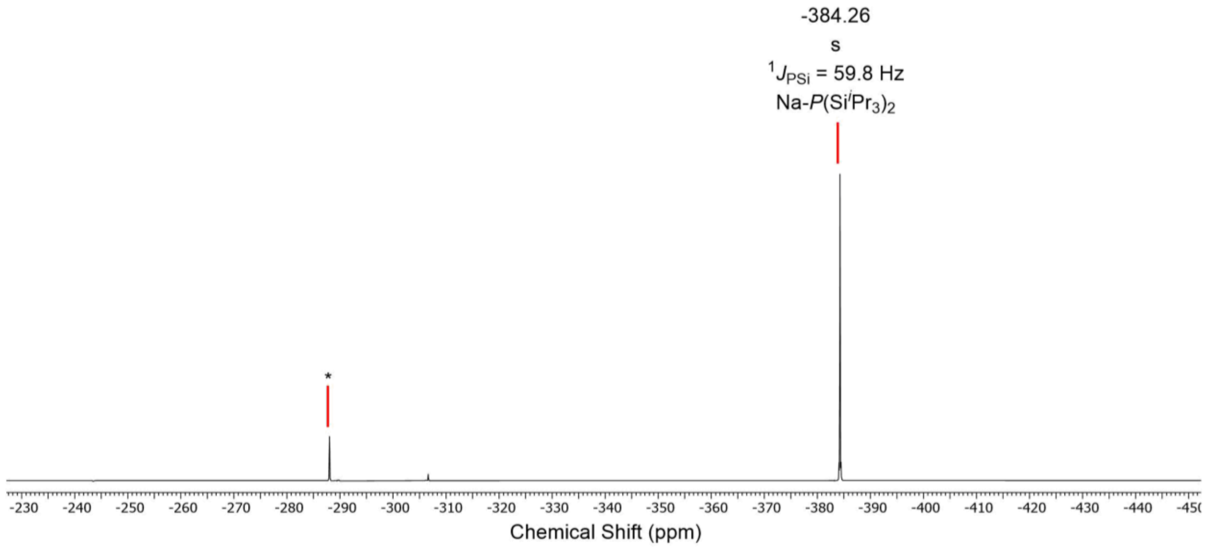

Figure S11. ^{31}P{^{1}H} NMR spectrum (162 MHz) of [Na{P(Si^{i}Pr$_3$)$_2$}] in d_8-THF. * denotes an impurity.

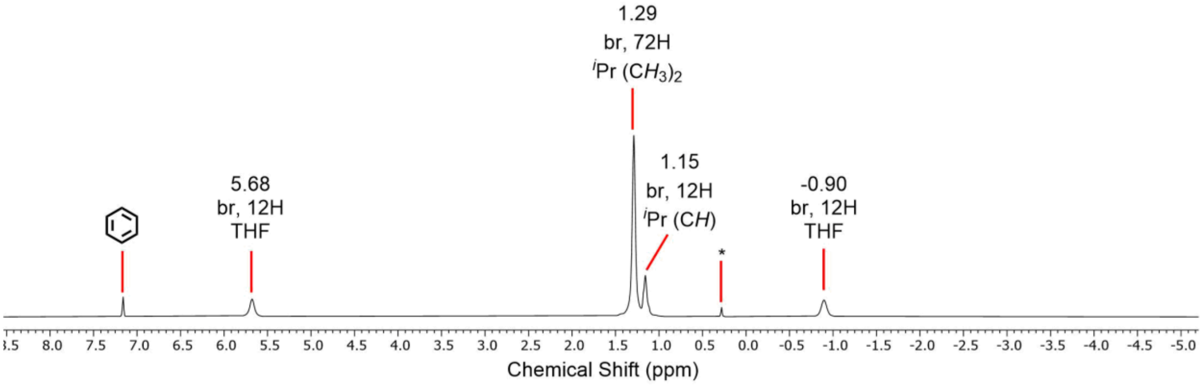

Figure S12. ^{1}H NMR spectrum (400 MHz) of **1-Sm** in C_6D_6. * denotes a diamagnetic impurity.

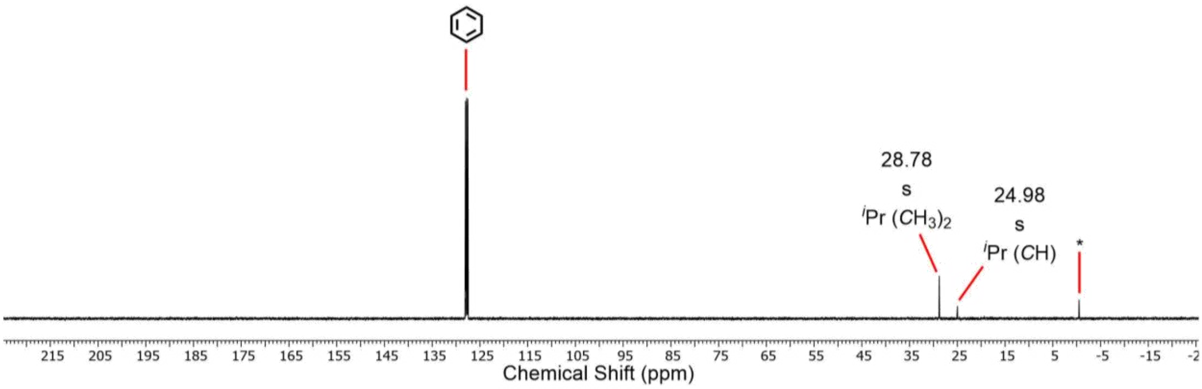

Figure S13. $^{13}C\{^1H\}$ NMR spectrum (101 MHz) of **1-Sm** in C_6D_6. * denotes silicon grease impurity.

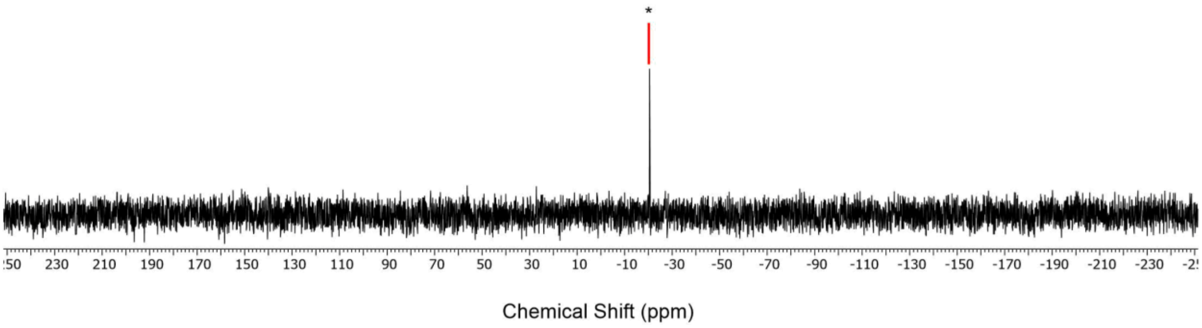

Figure S14. ^{29}Si DEPT90 NMR spectrum (79 MHz) of **1-Sm** in C_6D_6. * denotes a diamagnetic impurity.

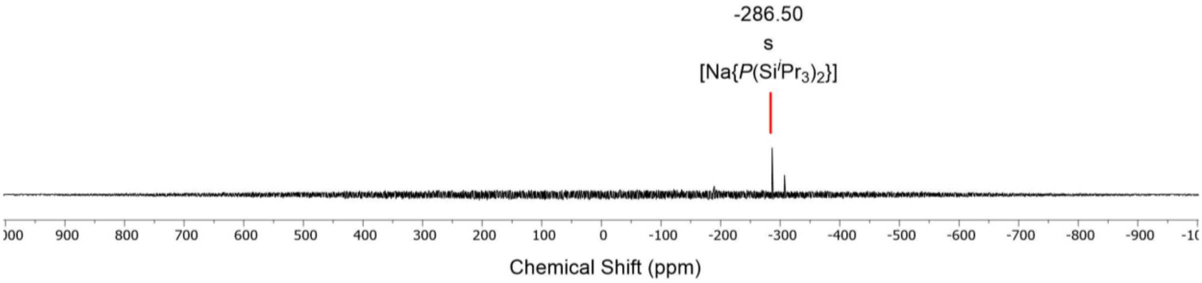

Figure S15. $^{31}P\{^1H\}$ NMR spectrum (162 MHz) of **1-Sm** in C_6D_6.

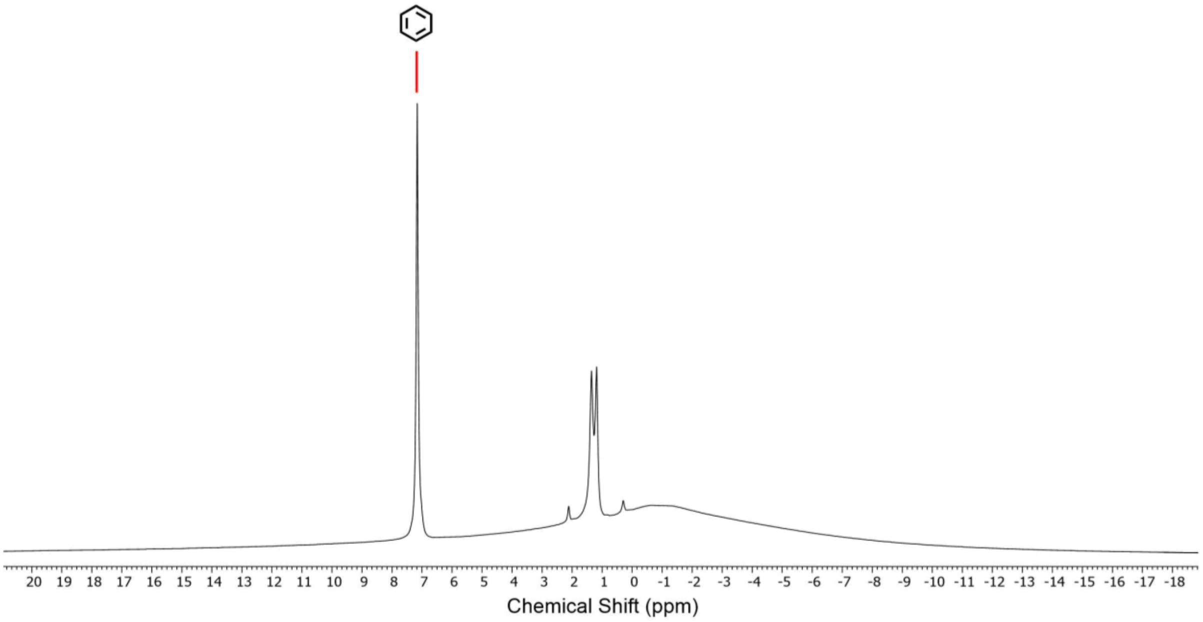

Figure S16. ^{1}H NMR spectrum (400 MHz) of **1-Eu** in C_6D_6. Expansion of spectrum of spectral width -100–100 ppm.

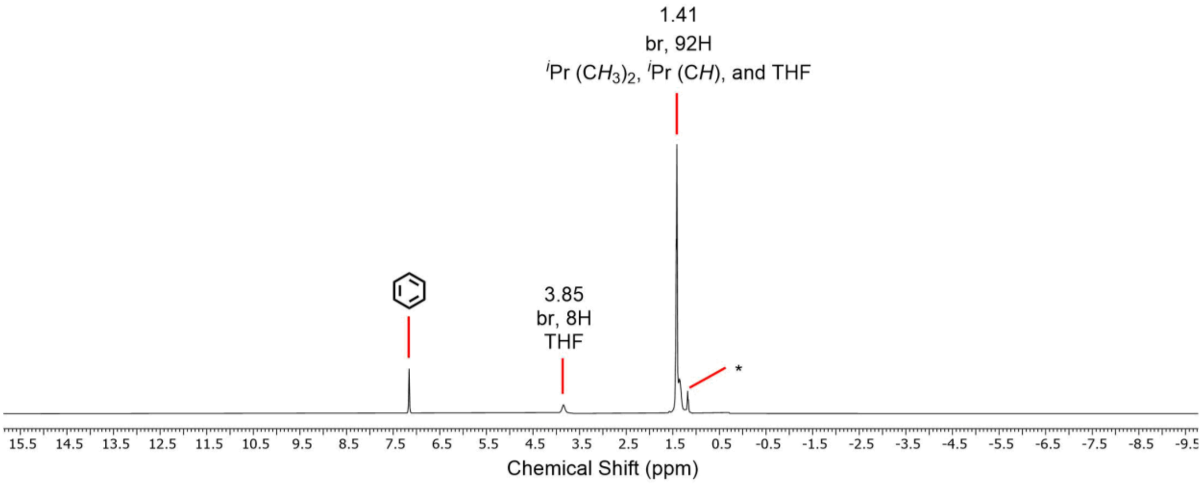

Figure S17. ^{1}H NMR spectrum (400 MHz) of **1-Yb** in C_6D_6. * denotes diamagnetic impurity.

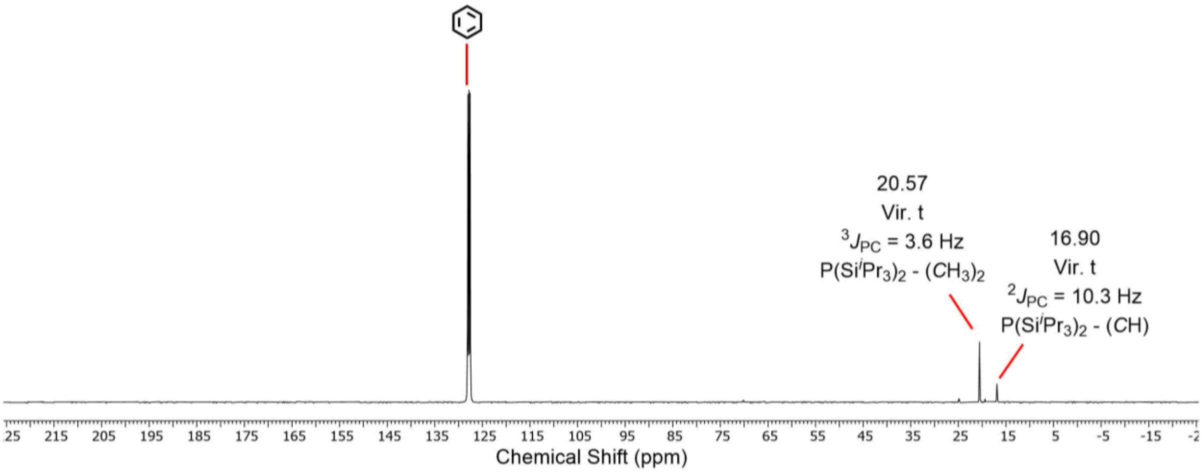

Figure S18. $^{13}C\{^1H\}$ NMR spectrum (101 MHz) of **1-Yb** in C_6D_6.

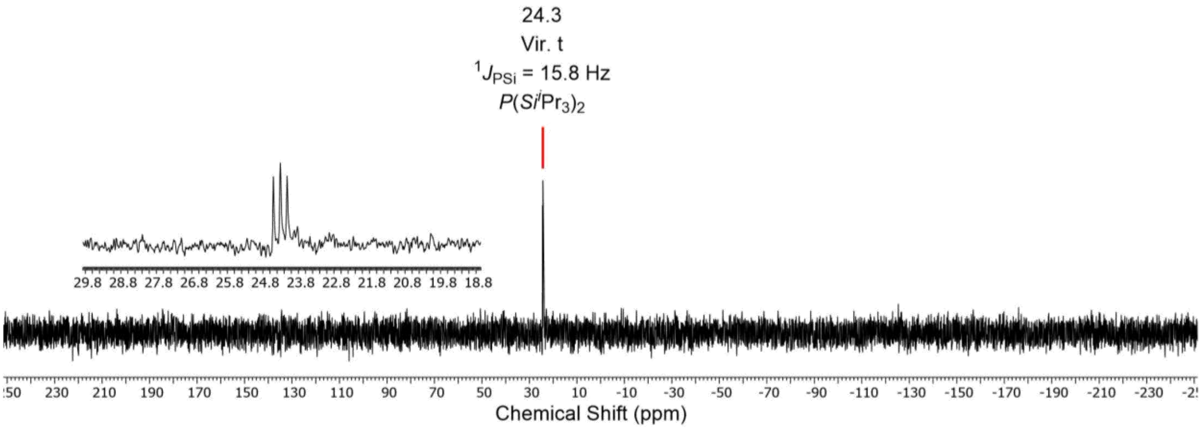

Figure S19. ^{29}Si DEPT90 NMR spectrum (79 MHz) of **1-Yb** in C_6D_6.

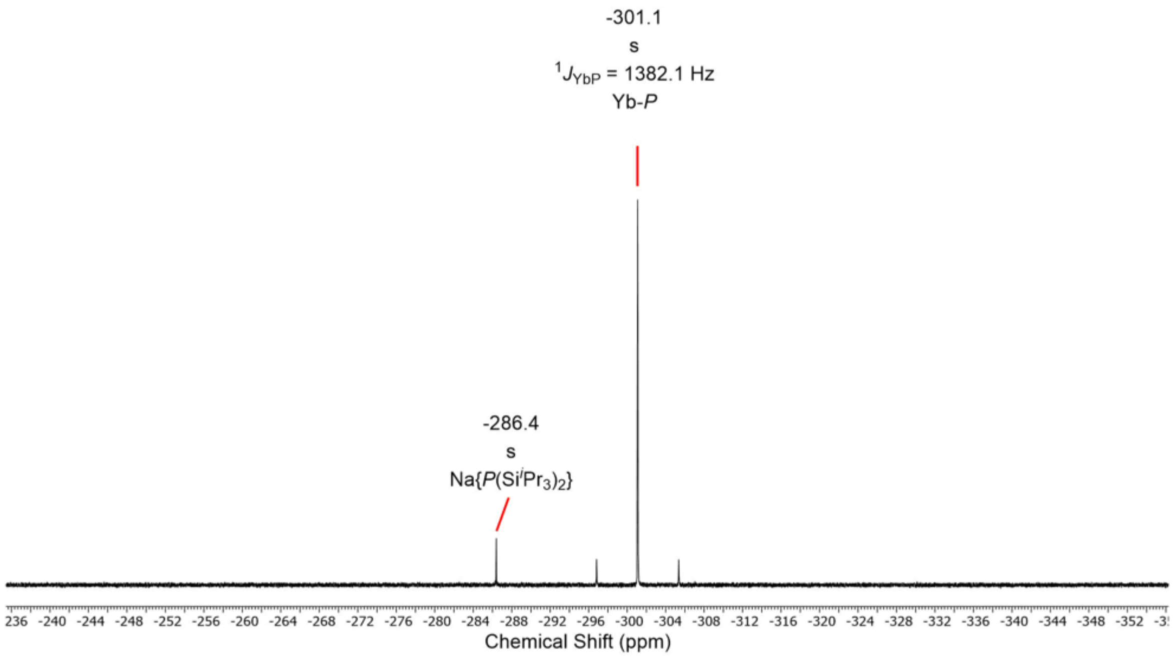

Figure S20. $^{31}P\{^{1}H\}$ NMR spectrum (162 MHz) of **1-Yb** in C_6D_6.

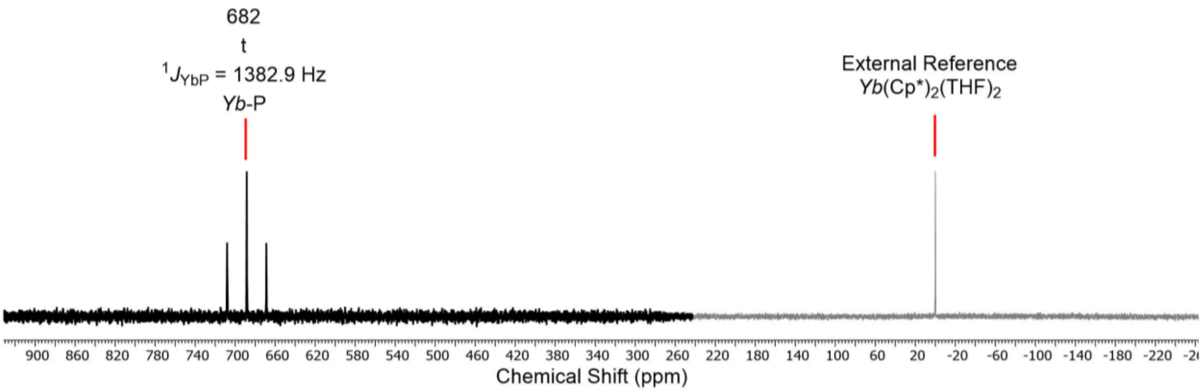

Figure S21. $^{171}Yb\{^{1}H\}$ NMR spectrum (71 MHz) of **1-Yb** in C_6D_6. $[Yb(Cp^*)_2(THF)_2]$ reference shown in order to show relative chemical shift.

5. Electronic UV-Vis-NIR absorption Spectroscopy

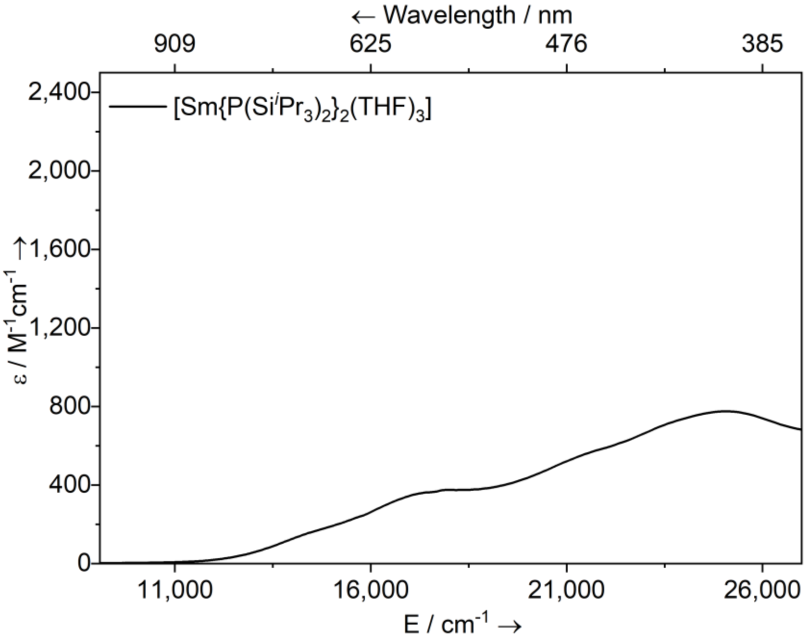

Figure S22. Electronic UV-Vis-NIR absorption spectra of **1-Sm** in toluene (2 mM) between 9091–27000 cm^{-1} (1100–370 nm).

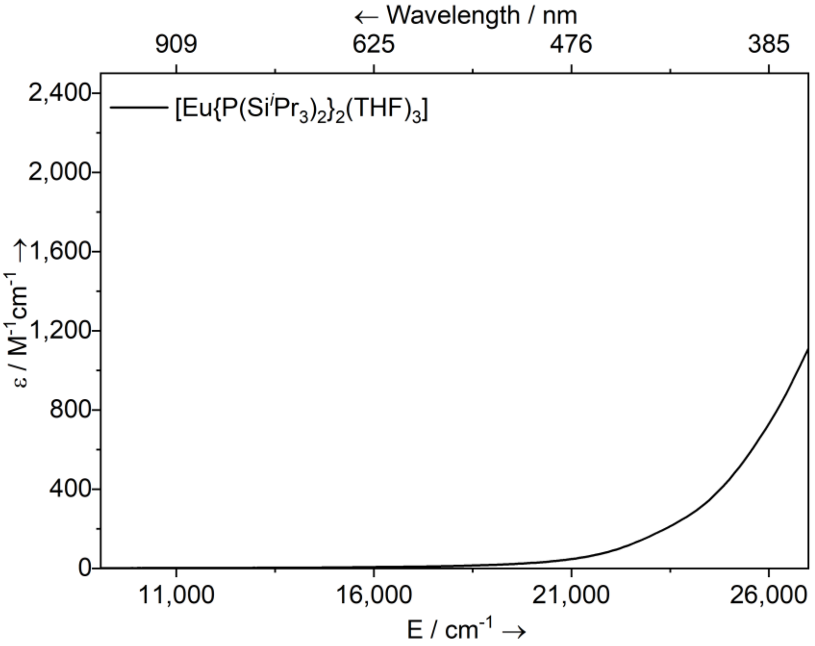

Figure S23. Electronic UV-Vis-NIR absorption spectra of **1-Eu** in toluene (2 mM) between 9091–27000 cm^{-1} (1100– 370 nm).

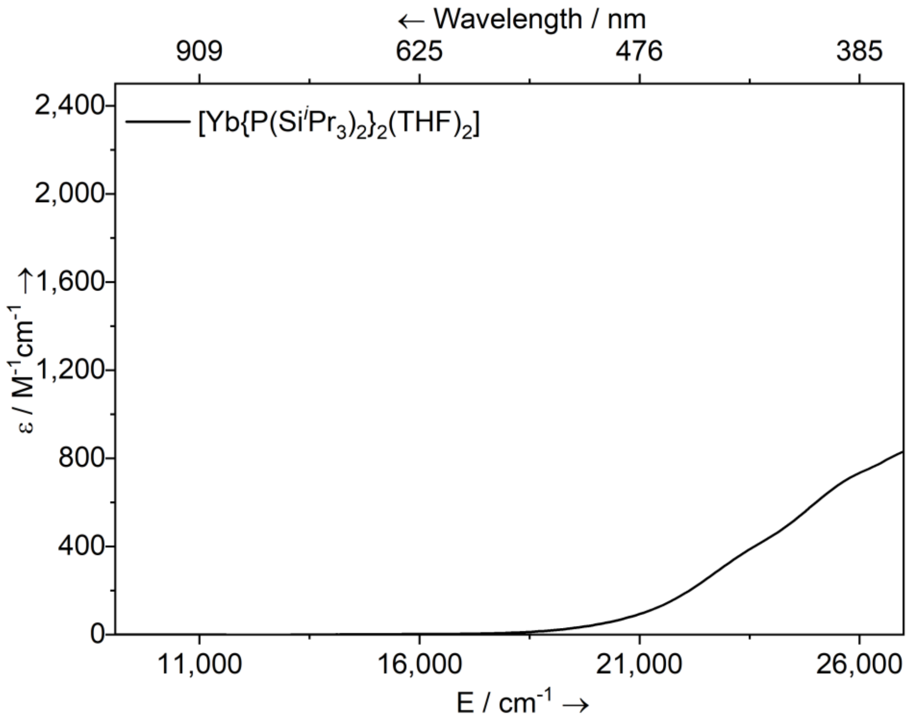

Figure S24. Electronic UV-Vis-NIR absorption spectra of **1-Yb** in toluene (2 mM) between 9091–27000 cm^{-1} (1100– 370 nm).

6. Photoluminescence Properties: 1-Eu, 1-Yb

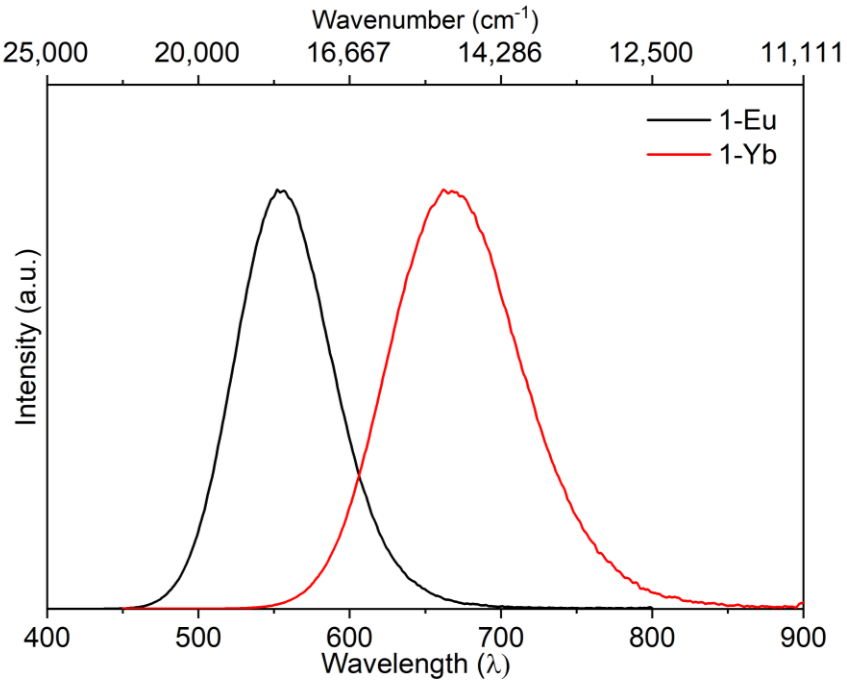

Figure S25. Normalized emission (**Em**) spectra of complexes **1-Eu** (black trace) and **1-Yb** (red trace) in toluene; **1-Eu** in toluene (2.01 mM), room temperature. Excited at 300 nm (Ex), observed at 555 nm (Em) and **1-Yb** in toluene (0.018 mM), room temperature. Excited at 300 nm (Ex), observed at 666 nm (Em).

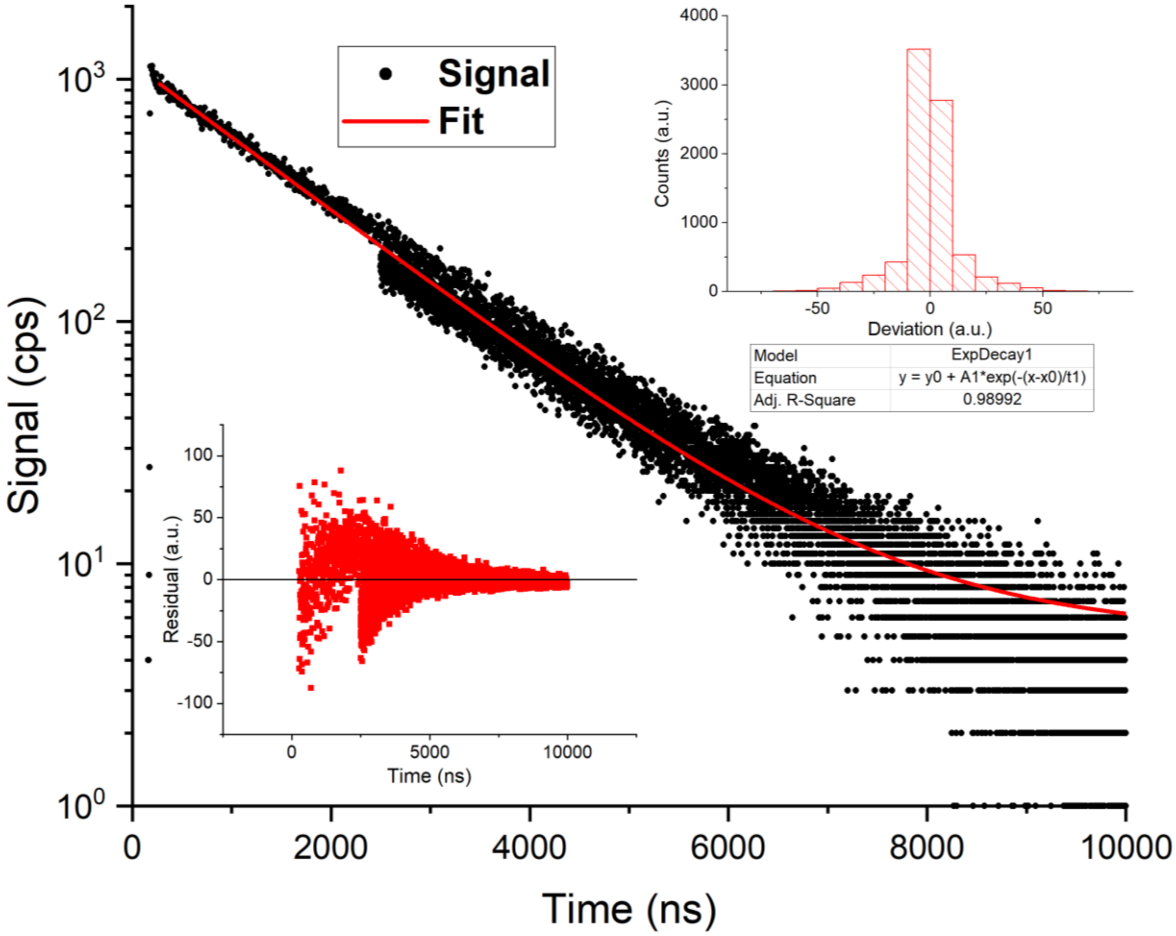

Figure S26. Fitted luminescence decay profile (black) of the emission signal in **1-Eu** with corresponding mono-exponential decay fit (red) (λex = 300 nm, λem = 555 nm, room temperature). Inset: associated residuals of the fit showing both normal distribution (bottom left) and expected error variance (top right).

7. SQUID Magnetometry: 1-Sm, 1-Eu

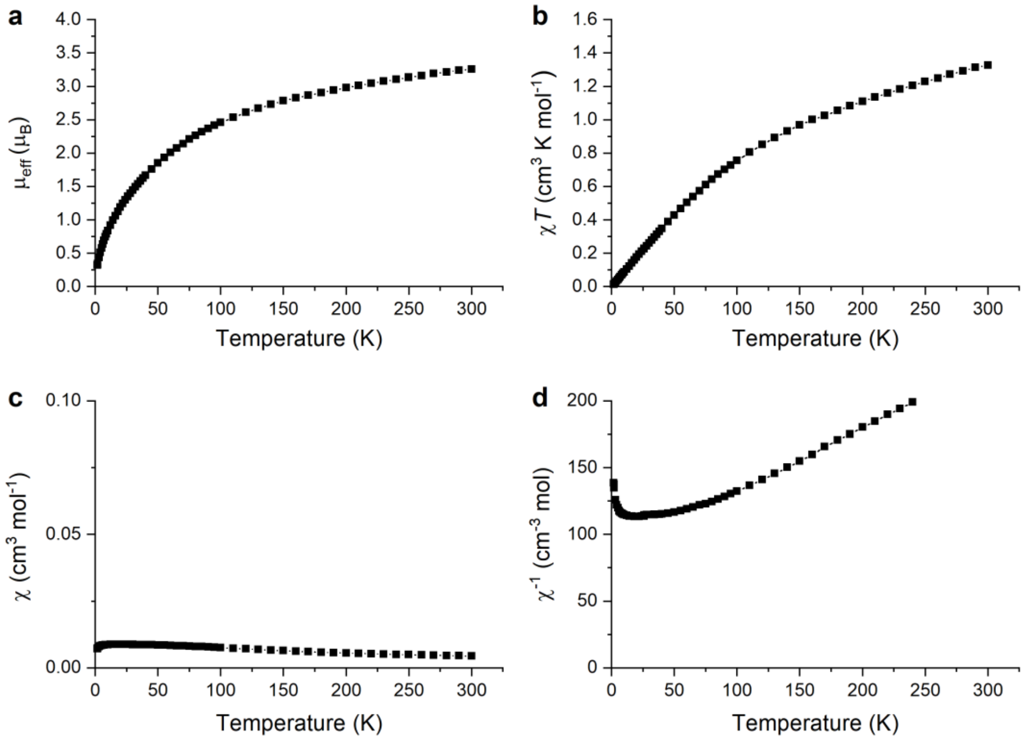

Figure S27. Variable-temperature SQUID magnetic data for powdered **1-Sm** in a 0.1 T applied magnetic field, presented as: **a** – μ_{eff} *vs*. T; **b** – χT *vs*. T; **c** – χ *vs*. T; **d** – χ^{-1} *vs*. T.

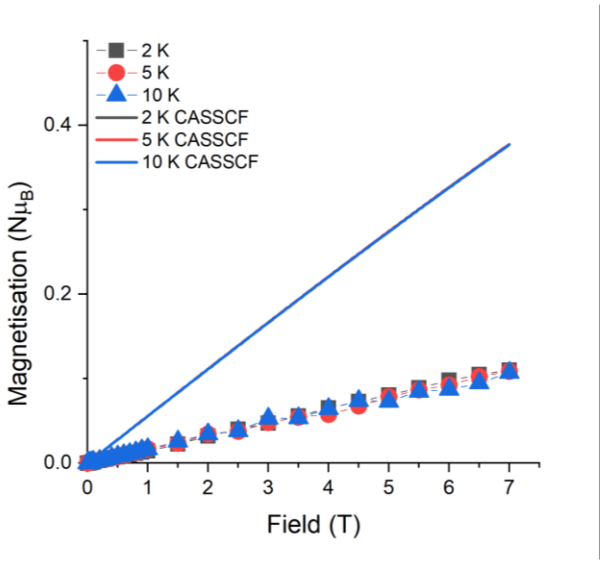

Figure S28. Magnetization *vs*. field plot for **1-Sm**. Solid lines show CASSCF results.

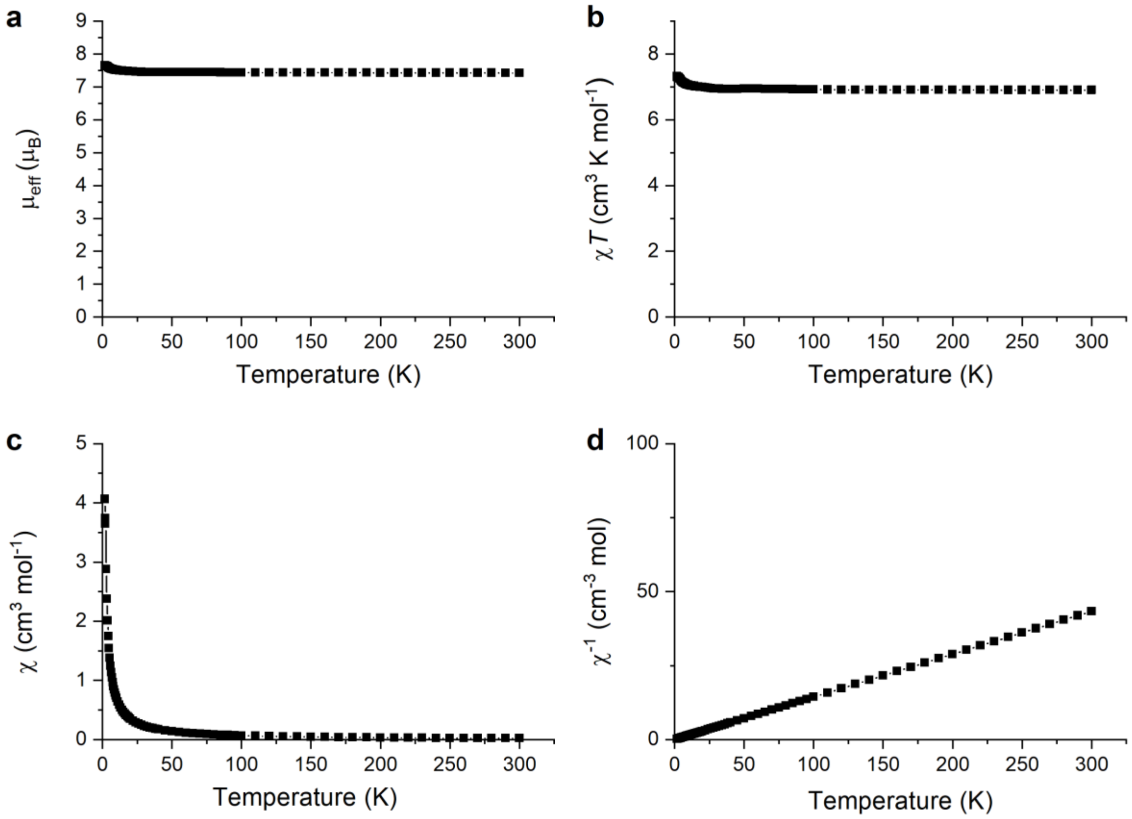

Figure S29. Variable-temperature SQUID magnetic data for powdered **1-Eu** in a 0.1 T applied magnetic field, presented as: **a** – μ_{eff} *vs*. T; **b** – χT *vs*. T; **c** – χ *vs*. T; **d** – χ^{-1} *vs*. T.

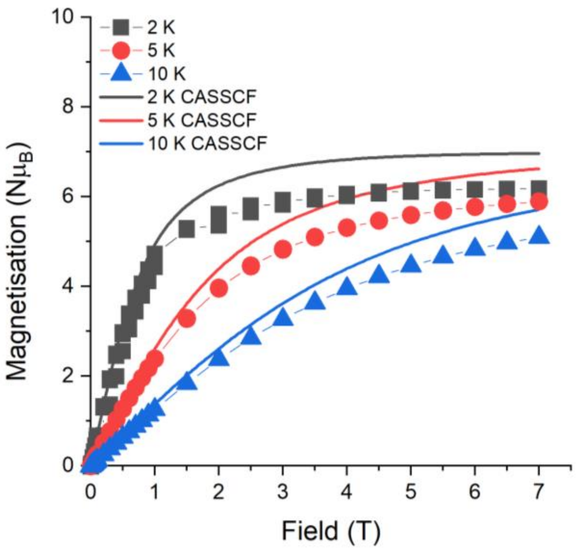

Figure S30. Magnetization *vs*. field plot for **1-Eu**. Solid lines show CASSCF results.

8. EPR Spectroscopy: 1-Eu

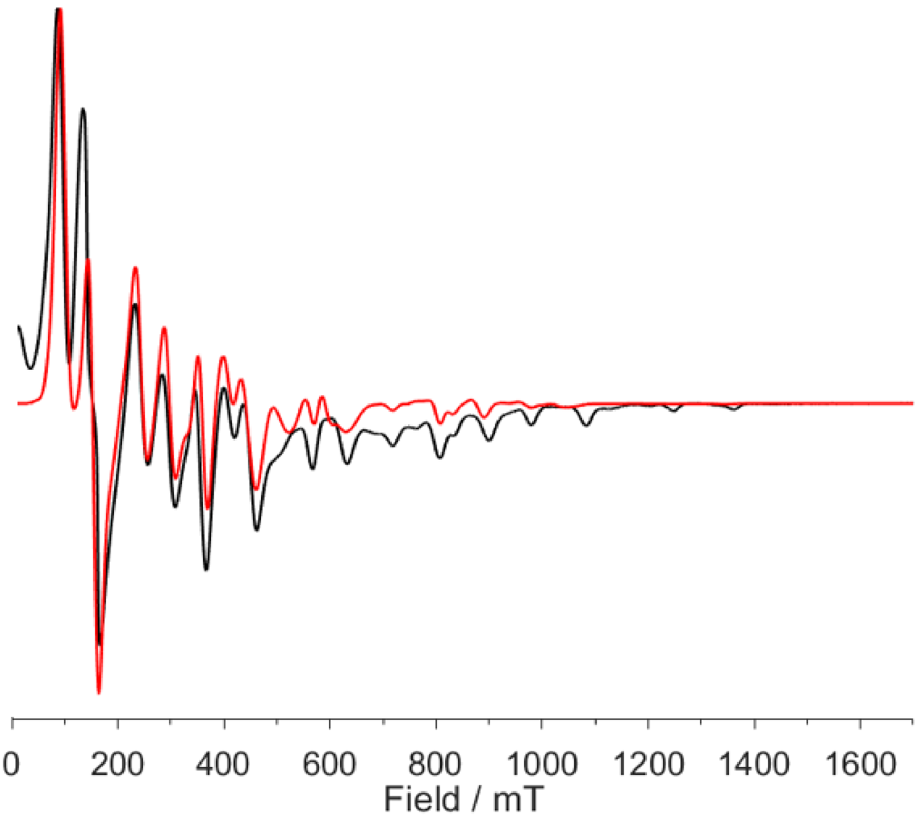

Figure S31. Experimental (black trace) and simulated (red trace) X-band powder EPR spectra for **1-Eu** at 5 K.

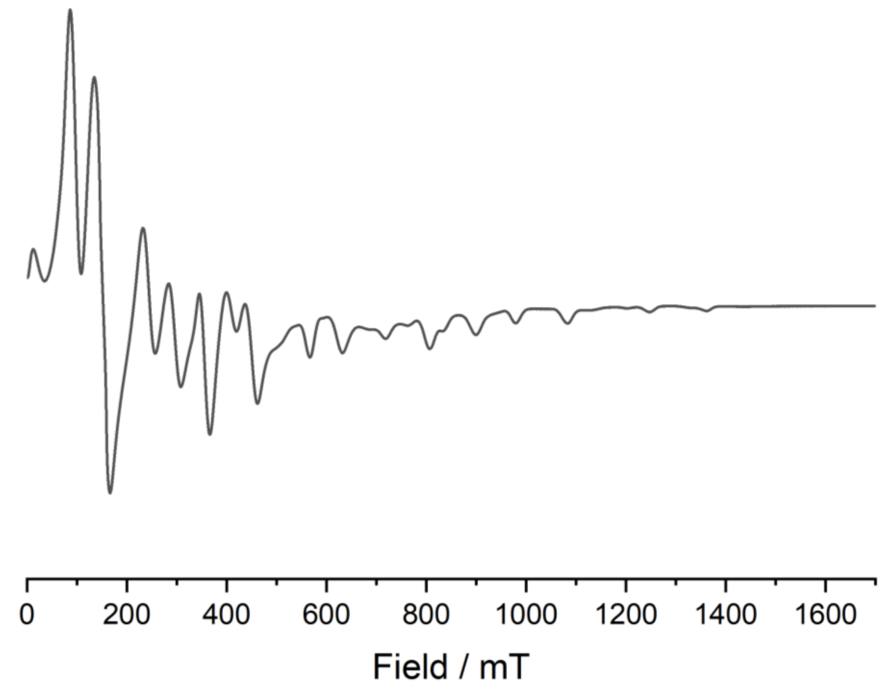

Figure S32. Powder X-band CW EPR spectrum of **1-Eu** measured at 10 K.

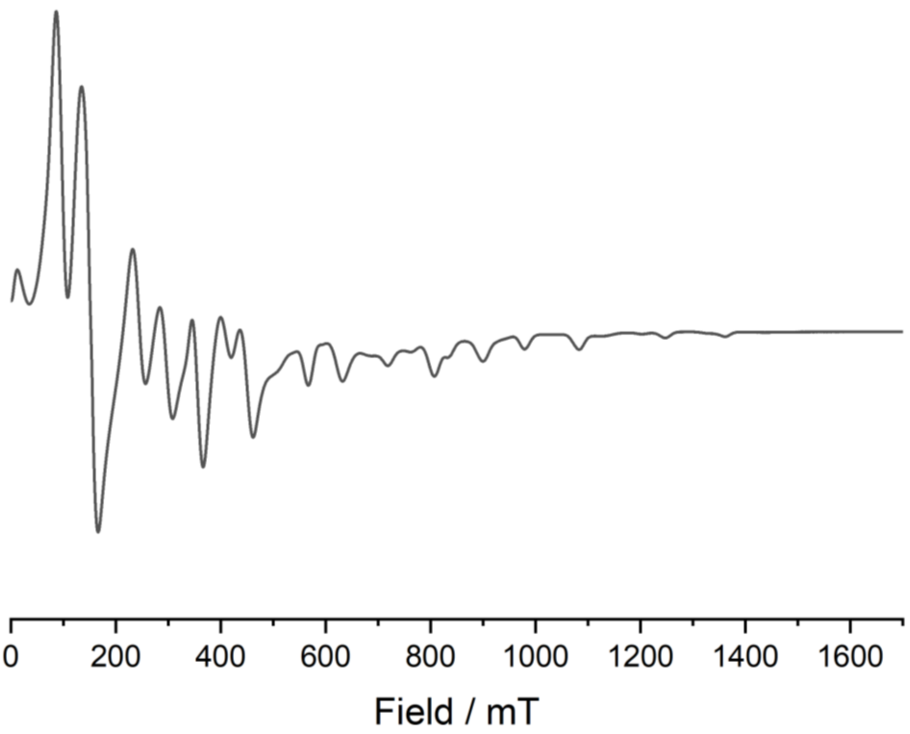

Figure S33. Powder X-band CW EPR spectrum of **1-Eu** measured at 20 K.

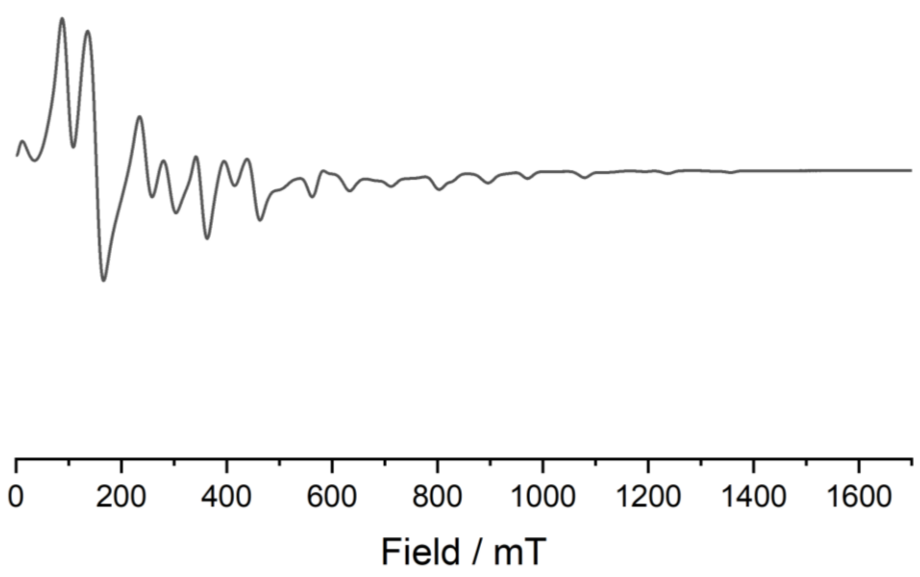

Figure S34. Powder X-band CW EPR spectrum of **1-Eu** measured at 50 K.

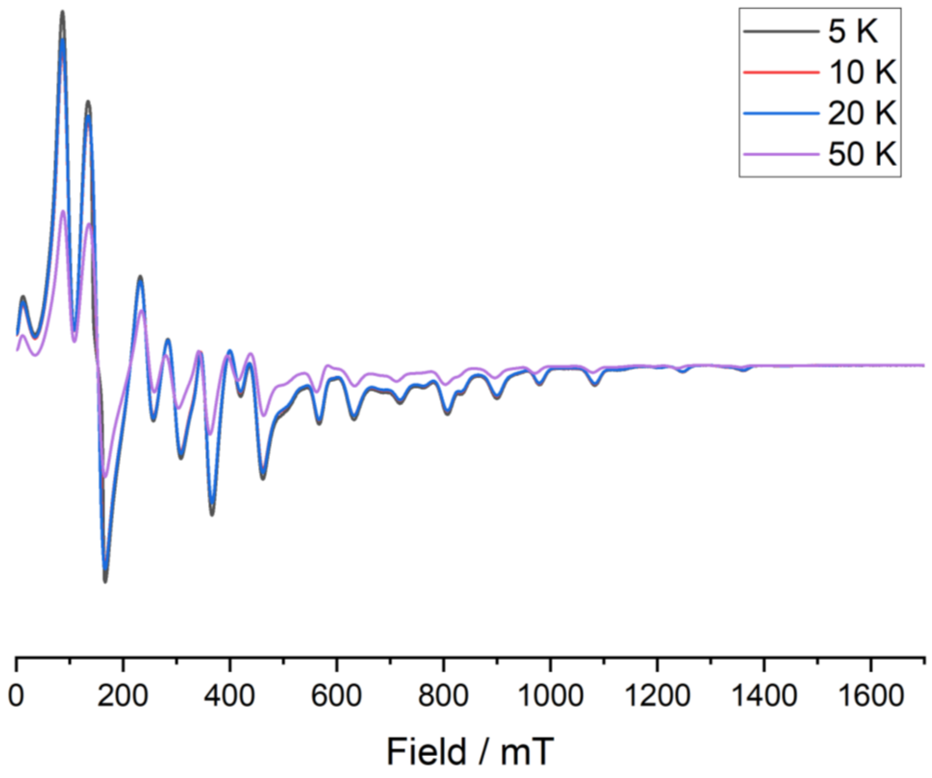

Figure S35. Overlaid powder X-band CW EPR spectra of **1-Eu** measured between 5-50 K.

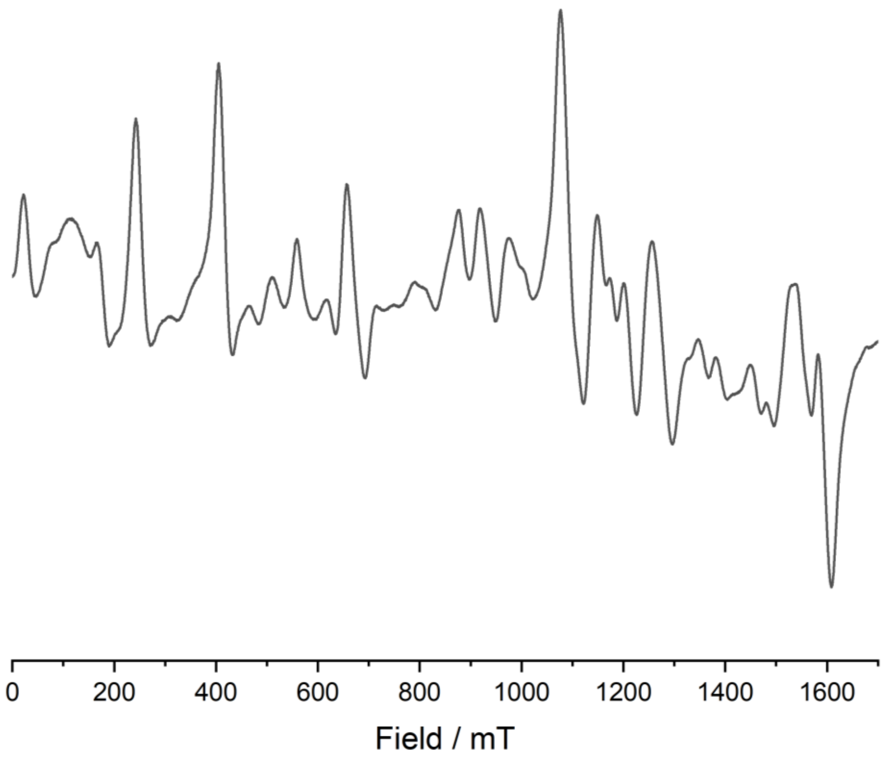

Figure S36. Powder Q-band CW EPR spectrum of **1-Eu** measured at 5 K.

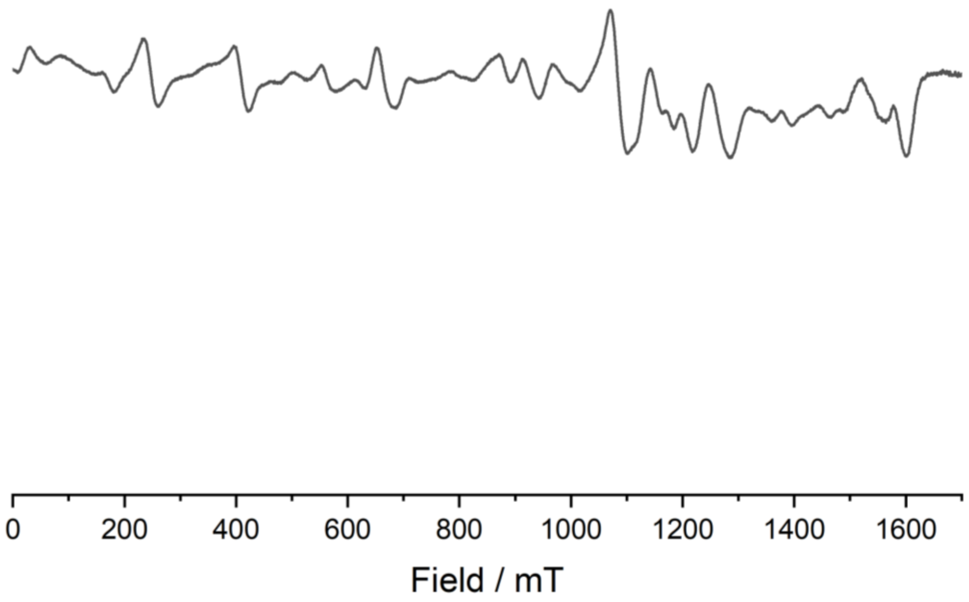

Figure S37. Powder Q-band CW EPR spectrum of **1-Eu** measured at 10 K.

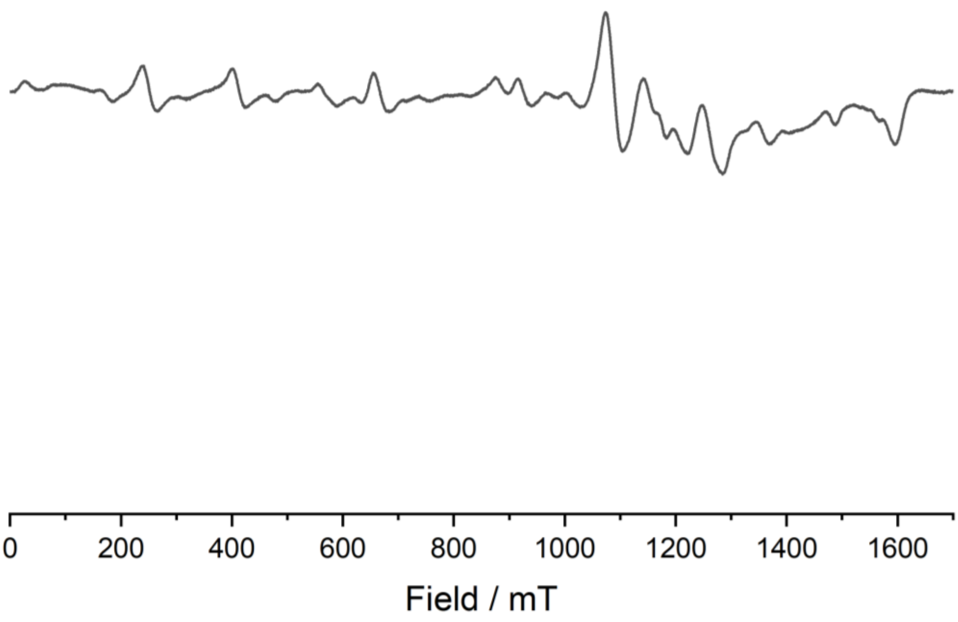

Figure S38. Powder Q-band CW EPR spectrum of **1-Eu** measured at 20 K.

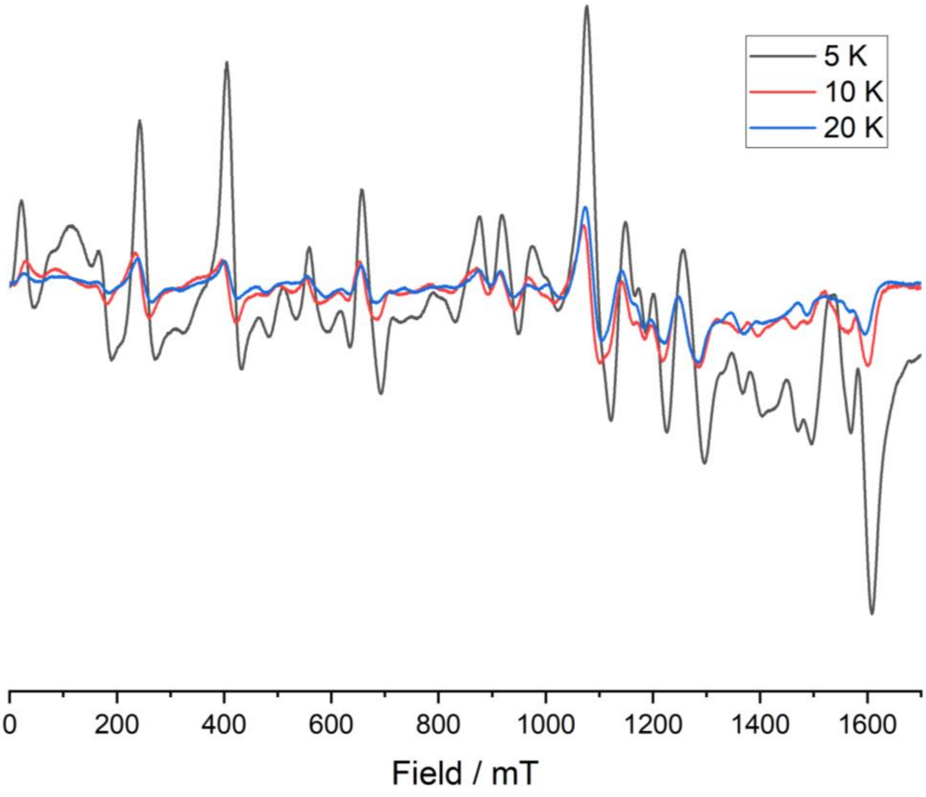

Figure S39. Overlaid powder Q-band CW EPR spectra of **1-Eu** measured between 5-20 K.

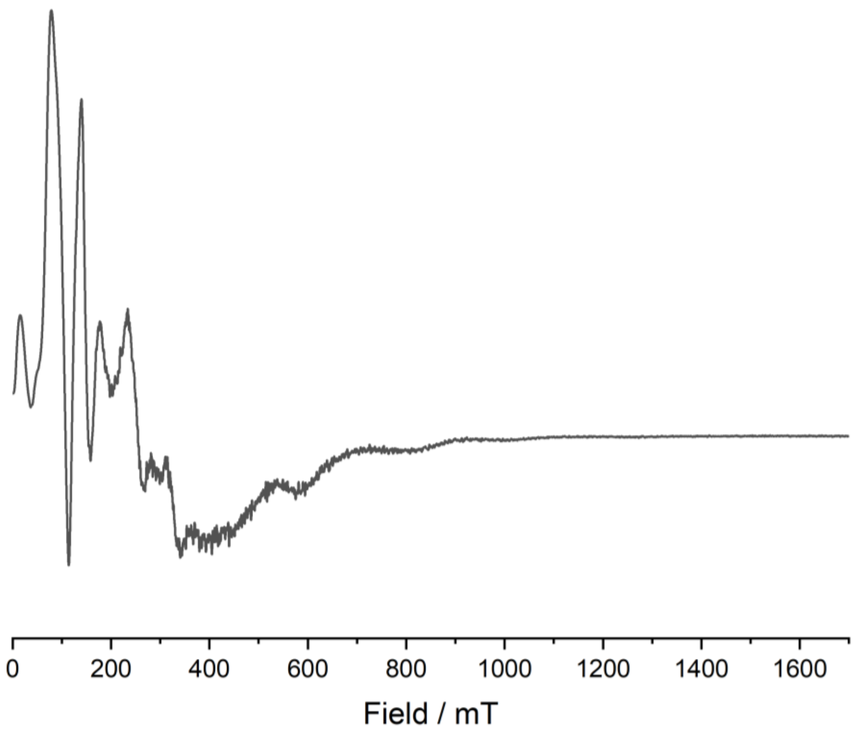

Figure S40. Frozen solution (10 mM) X-band CW EPR spectrum of **1-Eu** (9:1, toluene:hexane) measured at 5 K.

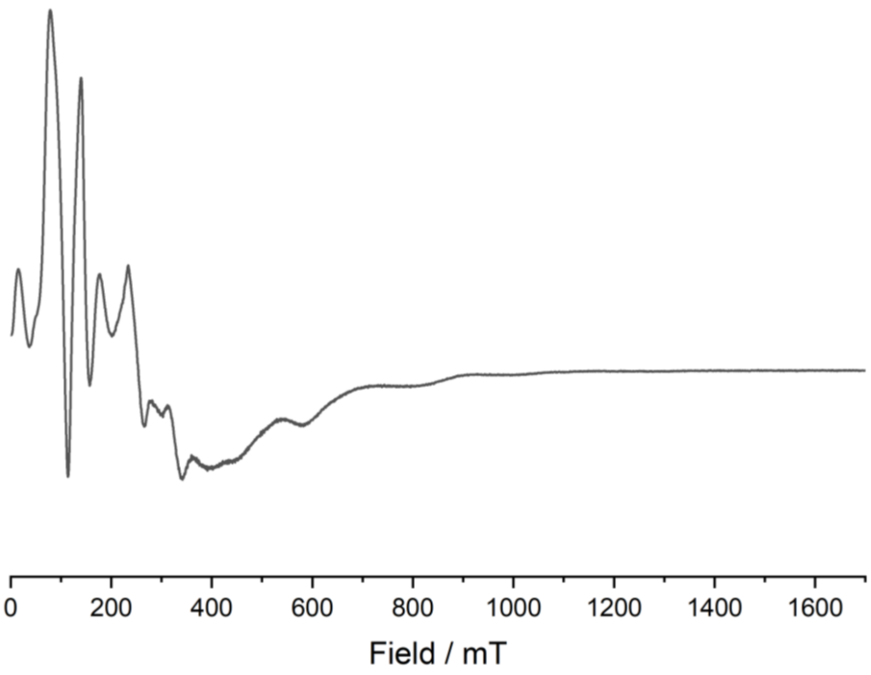

Figure S41. Frozen solution (10 mM) X-band CW EPR spectrum of **1-Eu** (9:1, toluene:hexane) measured at 10 K.

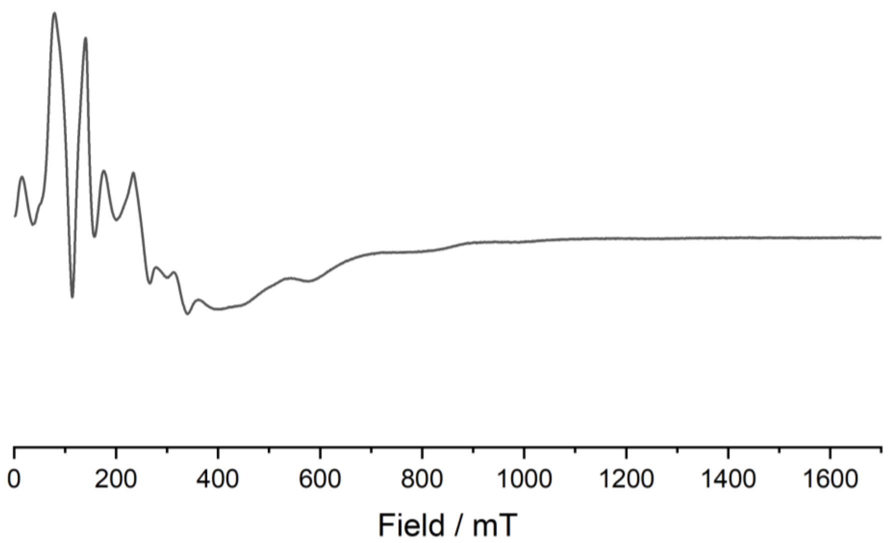

Figure S42. Frozen solution (10 mM) X-band CW EPR spectrum of **1-Eu** (9:1, toluene:hexane) measured at 20 K.

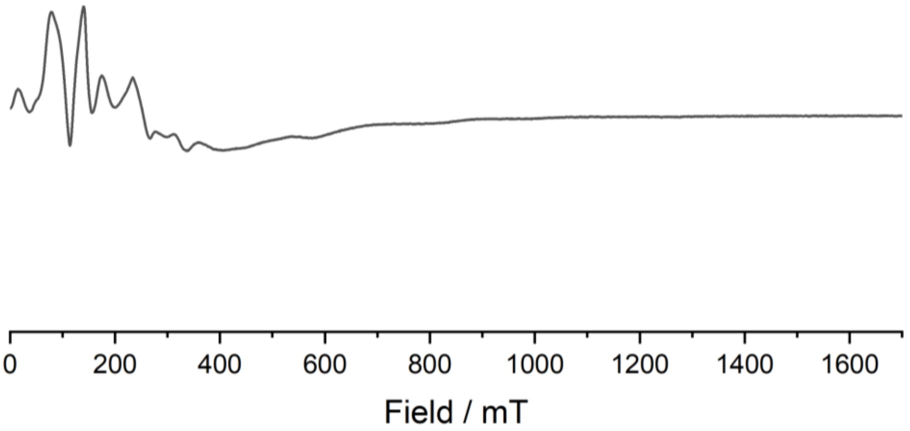

Figure S43. Frozen solution (10 mM) X-band CW EPR spectrum of **1-Eu** (9:1, toluene:hexane) measured at 50 K.

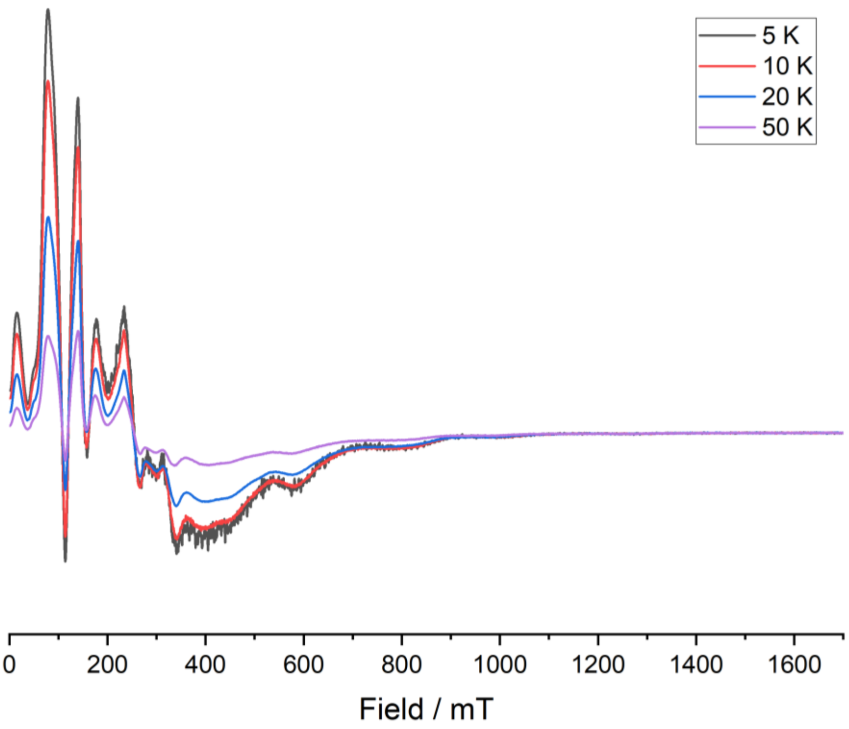

Figure S44. Overlaid frozen solution (10 mM) X-band CW EPR spectra of **1-Eu** measured between 5-50 K.

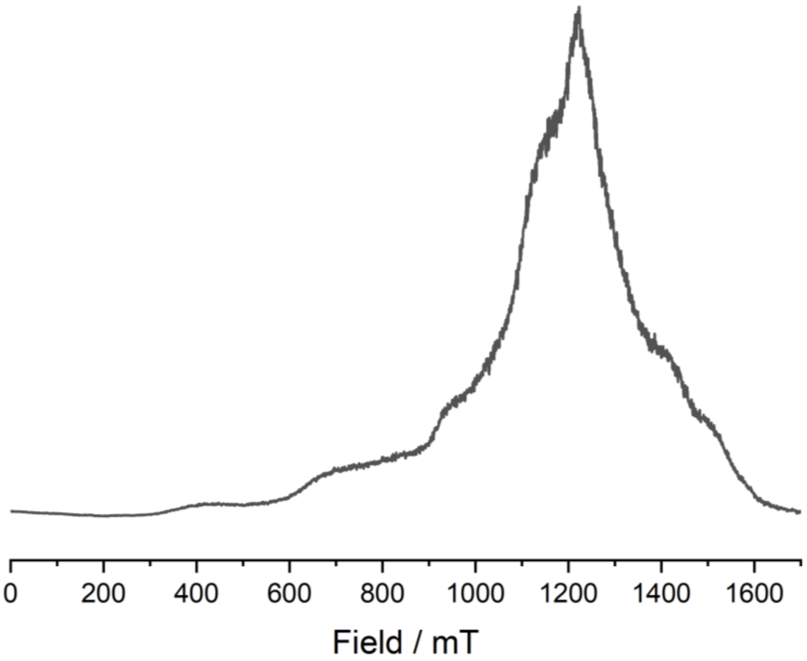

Figure S45. Frozen solution (10 mM) Q-band CW EPR spectrum of **1-Eu** (9:1, toluene:hexane) measured at 5 K.

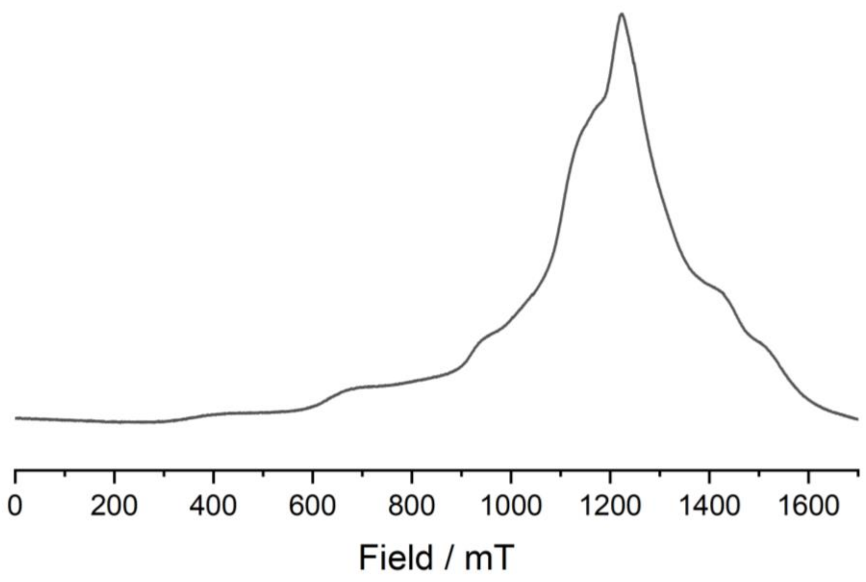

Figure S46. Frozen solution (10 mM) Q-band CW EPR spectrum of **1-Eu** (9:1, toluene:hexane) measured at 10 K.

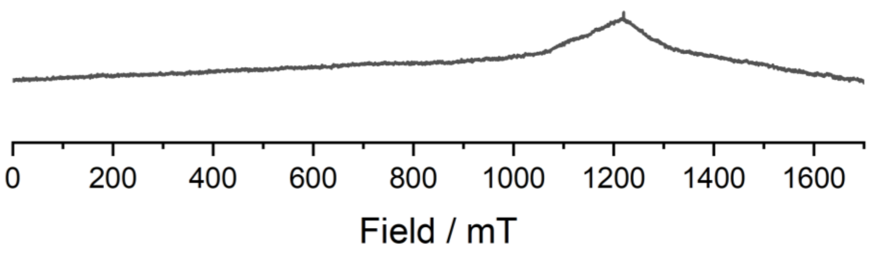

Figure S47. Frozen solution (10 mM) Q-band CW EPR spectrum of **1-Eu** (9:1, toluene:hexane) measured at 20 K.

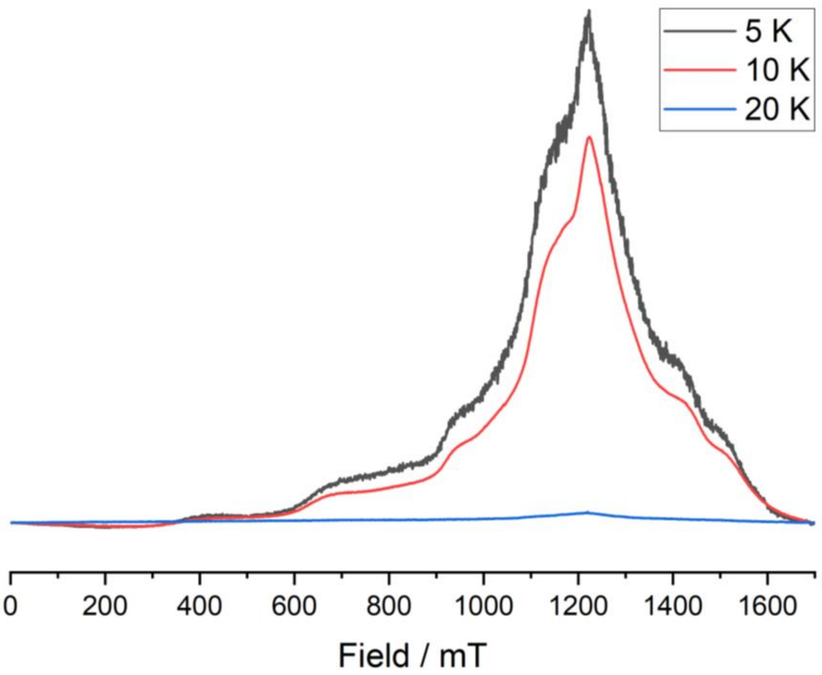

Figure S48. Overlaid frozen solution (10 mM) Q-band CW EPR spectra of **1-Eu** measured between 5-20 K.

9. *Ab initio* calculations: 1-Sm, 1-Eu

Table S6. Electronic structure of **1-Sm** obtained from CASSCF-SO using the solid-state geometry of **1-Sm**. States are non-degenerate. [a] Contributions > 2%.

Energy (cm^{-1})	Energy (K)	Wavefunction[a]
0	0	80%|0⟩
650.30	935.64	17%|0⟩

Table S7. Electronic structure of **1-Eu** obtained from CASSCF-SO using the solid-state geometry of **1-Eu**. Each row corresponds to a Kramers doublet. [a] Contributions > 2%.

Energy (cm^{-1})	Energy (K)	Wavefunction[a]	$<J_z>$
0	0	41%|± 1/2⟩ + 39%|± 3/2⟩ + 11%|∓ 5/2⟩ + 6%|± 1/2⟩	±1.09
0.263	0.378	39%|± 1/2⟩ + 24%|± 5/2⟩ + 23%|± 3/2⟩ + 8%|∓ 1/2⟩	±1.24
0.649	0.934	41%|± 5/2⟩ + 33%|± 3/2⟩ + 20%|∓ 7/2⟩	±2.28
1.241	1.786	74%|± 7/2⟩ + 22%|± 5/2⟩	±3.23

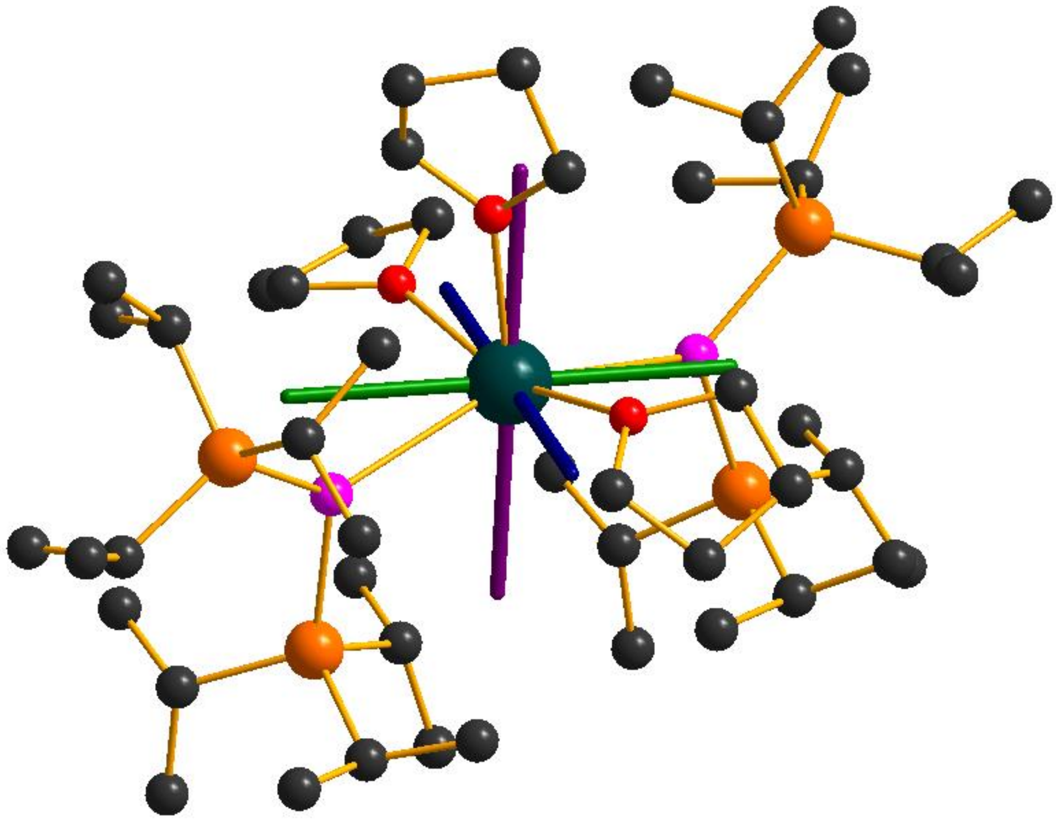

Figure S49. CASSCF-calculated magnetic axes for ground doublet of **1-Eu** (blue: g_1, violet: g_3, green: g_2,). Lanthanide, phosphorus, silicon, oxygen and carbon shown as metallic green, magenta, orange, red and grey respectively. Hydrogen atoms omitted for clarity.

10. DFT calculations: 1-Yb

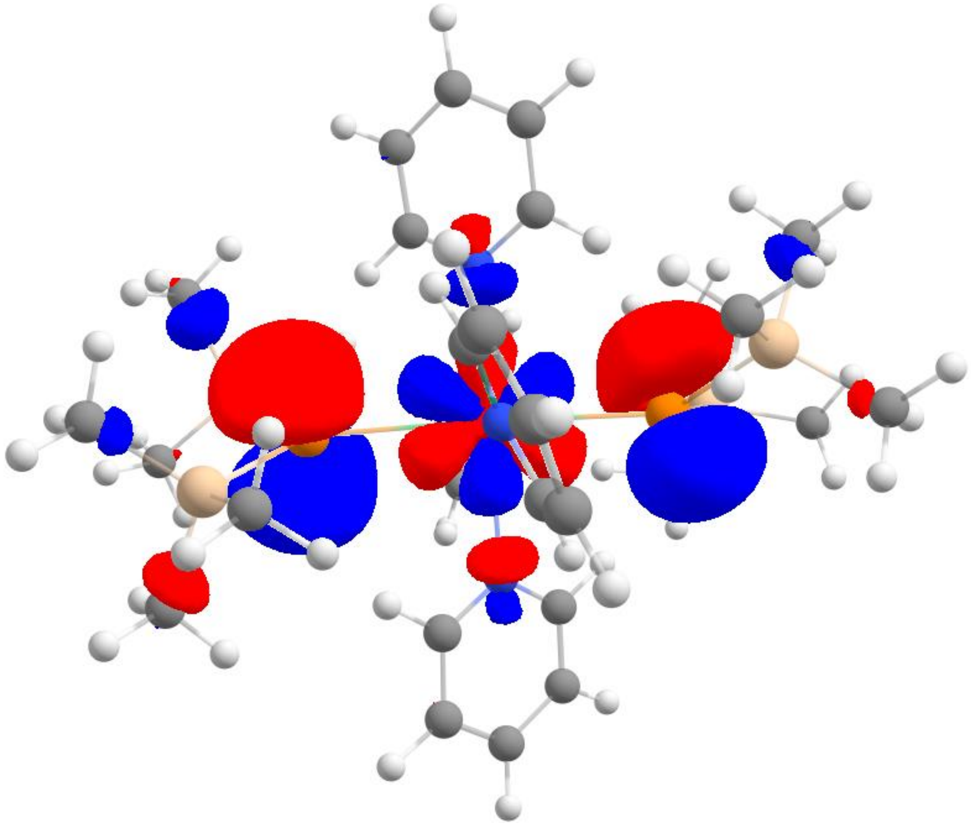

Figure S50. The highest occupied molecular orbital (HOMO) of **1-Yb** visualized at a 0.01 a.u. isosurface value.
